# Dysregulation of the endoplasmic reticulum blocks recruitment of centrosome-associated proteins resulting in mitotic failure

**DOI:** 10.1242/dev.201917

**Published:** 2023-11-16

**Authors:** Katherine R. Rollins, J. Todd Blankenship

**Affiliations:** Department of Biological Sciences, University of Denver, Denver, CO 80208, USA

**Keywords:** Rab proteins, Rab1, Endoplasmic reticulum, Mitotic spindle, Syncytial divisions

## Abstract

The endoplasmic reticulum (ER) undergoes a remarkable transition in morphology during cell division to aid in the proper portioning of the ER. However, whether changes in ER behaviors modulate mitotic events is less clear. Like many animal embryos, the early *Drosophila* embryo undergoes rapid cleavage cycles in a lipid-rich environment. Here, we show that mitotic spindle formation, centrosomal maturation, and ER condensation occur with similar time frames in the early syncytium. In a screen for Rab family GTPases that display dynamic function at these stages, we identified Rab1. *Rab1* disruption led to an enhanced buildup of ER at the spindle poles and produced an intriguing ‘mini-spindle’ phenotype. ER accumulation around the mitotic space negatively correlates with spindle length/intensity. Importantly, centrosomal maturation is defective in these embryos, as mitotic recruitment of key centrosomal proteins is weakened after *Rab1* disruption. Finally, division failures and ER overaccumulation is rescued by Dynein inhibition, demonstrating that Dynein is essential for ER spindle recruitment. These results reveal that ER levels must be carefully tuned during mitotic processes to ensure proper assembly of the division machinery.

## Teaser

**Summary:** In *Drosophila*, centrosomal maturation and endoplasmic reticulum condensation occur with similar timings at mitotic onset, and endoplasmic reticulum dysregulation leads to spindle weakening and failures of mitosis.

## INTRODUCTION

Mitosis is a highly dynamic process that requires pronounced morphological changes of various subcellular components and organelles. The endoplasmic reticulum (ER) membrane system displays intriguing behaviors throughout cell division, but the purpose of its dramatic reorganization remains enigmatic. In multiple model systems, the mitotic ER has been shown to reorganize, demonstrating changes in the relative amounts of cisternal and tubular structures (Puhka et al., 2012; Wang et al., 2013) separation into large membrane pools (Lu et al., 2009), and/or the accumulation at spindle poles (Terasaki, 2000; Bobinnec et al., 2003). The ER's movements are often linked to microtubules and microtubule motor protein function in both mitotic and interphase phases of the cell cycle (Terasaki et al., 1986; Frescas et al., 2006; Wang et al., 2013; Smyth et al., 2015; Diaz et al., 2019). The mitotic shifts in ER populations during division appear to contribute to the equal partitioning and inheritance of the ER into daughter cells (reviewed by Du et al., 2004). Though this may explain a passive spindle pole association of the ER, it does not clarify whether a reciprocal relationship exists by which the ER may affect the formation and function of the mitotic spindle.

Two major mechanisms are generally present when cell or organellar morphologies rapidly shift during development: cytoskeletal force generation and membrane trafficking (Schejter and Wieschaus, 1993; LeGoff and Lecuit, 2015; Xie et al., 2018; Loerke and Blankenship, 2020). Indeed, in the early embryo the rapid remodeling of the plasma membrane during mitotic divisions has been shown to be mediated by both of these mechanisms (Foe and Alberts, 1983; Foe et al., 2000; Riggs et al., 2003; Fabrowski et al., 2013; Yan et al., 2013; Holly et al., 2015; Lee et al., 2015; Rodrigues et al., 2016; Mavor et al., 2016; Sherlekar and Rikhy, 2016; Zhang et al., 2018; Sherlekar et al., 2020; Xie et al., 2021; Henry et al., 2022; Miao et al., 2023). Because the ER is an organelle that is central to membrane trafficking, it is possible that changes in trafficking events during the cell cycle may also contribute to the overall shaping of the ER. Although most studies have focused on microtubule, ER-shaping protein, and motor protein contributions to ER morphologies during mitosis in the early embryo, (Smyth et al., 2015; Diaz et al., 2019; Araújo et al., 2022), less work has been done to elucidate whether ER shapes and remodeling during mitosis also depend on trafficking of membrane in or out of the ER. The Rab family of GTPase proteins are a large group of proteins that are key mediators of membrane trafficking. Rab proteins direct membrane traffic by recruiting effectors that guide the movement of vesicular and compartmental behaviors (Gibieža and Prekeris, 2017; Homma et al., 2021). Additionally, Rabs ensure the specificity of the target compartment by interacting with specific tethers and SNARE proteins (Grosshans et al., 2006). The Rab1 subfamily is one of only five conserved Rabs that can be found in organisms from yeast to humans (Homma et al., 2021) and has been well-characterized to mediate traffic between the ER and Golgi. Additionally, it has been suggested that Rab proteins at the ER may serve non-trafficking functions, such as forming organelle contacts and associating with shaping proteins, such as atlastin and reticulons (Sandoval and Simmen, 2012).

The *Drosophila* embryo provides an excellent model in which to study mitotic events *in vivo* owing to the ability to capture multiple rounds of division in a short period of time in an intact organism. Early in development, the embryo undergoes 13 rounds of nuclear division in a syncytium with transient membrane furrows. During cell cycles 1-9, nuclei slowly migrate from deep within the yolk to the outer periphery of the embryo where they complete cell cycles 10-13, also known as the cortical divisions. During these divisions, the nuclei become increasingly densely packed as they occupy a common subcortical plane, making the formation of membrane furrows necessary to prevent mitotic division failures (Foe and Alberts, 1983; Sullivan et al., 1993). Furrow defects can result in genetic instability through two major mechanisms: a spindle anchoring mechanism, whereby shortened furrows do not properly support adhesion of astral microtubules to the plasma membrane, and a spindle corralling mechanism in which defective furrows fail to separate adjacent chromosomal complements and daughter nuclei combine with one another (Holly et al., 2015). Mitotic defects through either of these mechanisms can be observed after the disruption of components of late exocytic trafficking machinery, such as Rab8, RalA or the exocyst subunits (Holly et al., 2015; Mavor et al., 2016). However, whether membrane-trafficking networks can directly impact mitotic formation and assembly during cleavage divisions is unclear.

Here, we use the *Drosophila* syncytial embryo to study the ER during cell divisions. The ER in the embryo is highly compartmentalized and remains associated with individual nuclei, despite these nuclei sharing cytoplasmic space (Frescas et al., 2006), and thus permits the study of the ER and division machinery as coupled units. We demonstrate that Rab1 is important for ER morphologies during mitosis and use *Rab1-*depleted embryos as a means to perturb ER dynamics to explore ER–spindle interactions. Disruption of *Rab1* during cleavage stages causes the shortening of mitotic spindles and division failures. Interestingly, *Rab1* depletion does not grossly affect furrow dynamics, bringing to light a new division failure mechanism in the embryo. These results indicate that spindle assembly depends on a well-regulated ER and provide insight into how dysregulation of the ER impacts the fidelity of cell divisions.

## RESULTS

### Mitotic ER morphologies are dependent on Rab1

**Fig. 1.**
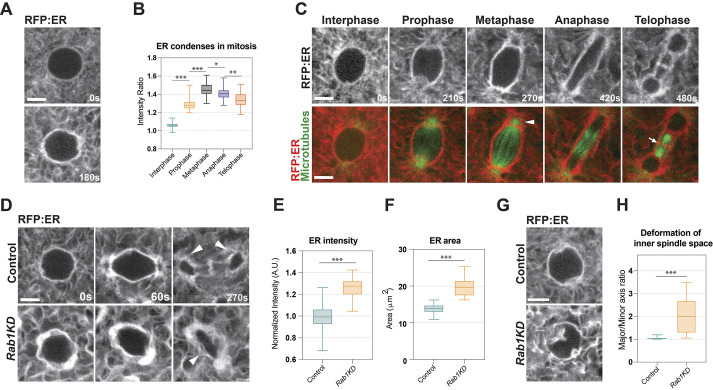
**The mitotic ER after *Rab1* perturbation.** (A) Still images from live imaging of the ER (UAS-RFP:ER) in interphase just prior to mitosis (0 s) and at the onset of mitosis (180 s) in cycle 11 embryos. (B) Quantification of ER spindle ‘coat’ intensity at different stages of mitosis based on the ratio of ER intensity at the interface of the inner spindle area and the intensity 2.5 µm external to the spindle zone. Cycle 11 embryos. k≥3, *n*=33. (C) Still images from live imaging of UAS-RFP:ER at different stages of mitosis along with a microtubule marker (endogenous Jupiter:GFP). Arrowhead indicates accumulation of ER near spindle pole in metaphase embryo; arrow indicates midbody accumulation of ER. (D) Still images from live imaging of UAS-RFP:ER in control and *Rab1* knockdown (shRNA; *Rab1KD*) embryos. Arrowheads in control panel indicate successful anaphase separation; arrowhead in *Rab1KD* panel indicates the continuance of a single, shared inner mitotic space. Images are optimized in order to compare morphologies; identically leveled images that demonstrate higher ER intensities in *Rab1* embryos are presented in Fig. S2A. (E,F) Quantification of ER accumulation at metaphase based on ER (RFP-ER) intensity (E) or area (F) in control and *Rab1*-disrupted embryos during cell cycle 11. k≥5, *n*≥44. (G) Still images from live imaging of UAS-RFP:ER in control and *Rab1-*depleted embryos in prophase where an invasion of the ER into the inner spindle space can be seen. (H) Quantification of ER deformation based on the ratio of two perpendicular axes of the inner spindle area at prophase in control versus *Rab1-*depleted embryos. k≥3, *n*≥25. All time points are relative to the initial image within the panel. *Rab1* depletion was achieved through shRNA2 (Fig. S1I). *P<0.05; **P<0.005; ***P<0.0005 (Mann–Whitney *U*-test). In box and whisker plots, box limits represent the inner quartile range, horizontal line represents the mean and whiskers the range. Scale bars: 5 µm. A.U., arbitrary units.

As a starting point to these studies, we wanted to characterize, by *in vivo* imaging, the morphological changes that the ER undergoes during the early cortical divisions in the *Drosophila* embryo. At the onset of mitosis, the ER appears to condense into more discrete tubular structures (Fig. 1A). This early ER transition results in enrichment of the ER in a region that appears to highlight the eventual location of the spindle, with ER intensities increased in prophase and metaphase by ∼50% then mildly decreasing later in the mitotic cycle (Fig. 1B). To examine how these changes in ER structures correlate with those of the microtubule spindle, we made use of a two-color imaging system in which microtubules (Jupiter:GFP) and ER (RFP:ER, through a KDEL tag) can be visualized together (Fig. 1C, Movie 1). These data revealed that changes in ER morphologies appear to be coordinated with those of the spindle and are consistent with previous studies that injected fluorescently labeled tubulin to visualize microtubule dynamics and/or used Pdi-GFP to mark the ER (Bobinnec et al., 2003; Bergman et al., 2015). At the beginning of spindle formation, as centrosomes begin to show the first signs of interpolar microtubule nucleation, the ER accumulated in a region around the centrosomes. This concentration of the ER around the spindle poles continued, with the ER-coated structure becoming further enriched; ER cradles surrounded the centrosomes while increasing in size (as measured by cross-sectional area; Fig. 1C, arrowhead). At anaphase, the ER appeared to elongate along with the spindles, facilitating sister chromosome separation. Finally, at telophase, the ER could be seen localized to a mid-body like region along with microtubules (Fig. 1C, arrow).

Given the close association of the ER with the mitotic spindle and the membrane-rich environment of the early embryo, we next wanted to examine how altering ER localization might affect syncytial mitoses. Previous work from our lab screened the Rab family of GTPases based on localization for proteins that are active in embryogenesis, but we did not observe Rab1 puncta or compartments at these stages (Jewett et al., 2017). However, this work relied on a semi-endogenous two-component system of Gal4 knock-ins at Rab loci driving UAS-YFP:Rab transgenes (Jin et al., 2012), which resulted in an imperfect detection of some Rab proteins. Using a new collection of endogenously expressed YFP:Rab protein lines (Dunst et al., 2015), we found that Rab1 was present in highly dynamic puncta in the early embryo (Fig. S1A). Rab1 is canonically appreciated to mediate ER to Golgi trafficking, and we observed a robust accumulation that was higher during the syncytial stages compared with later stages (Fig. S1B,C). We were therefore interested in what would happen to ER structures after disruption of *Rab1* function, and whether this could be a tool to explore the relationship between the ER and spindle formation during rapid cleavage divisions. Indeed, embryos expressing a fluorescent ER marker (RFP:ER) and compromised for *Rab1* function, through expression of a small hairpin RNA (shRNA) targeted to Rab1, revealed a deeply perturbed ER at multiple mitotic stages (Fig. 1D). Beginning at prophase and continuing through metaphase, ER membranes formed large accumulations and the overall amount of ER located near the mitotic space increased in both area and intensity in *Rab1*-disrupted embryos (Fig. 1D-F, Fig. S1D). Although the ER condensed in *Rab1*-depleted embryos at mitotic onset, the clear definition between the ER coat and the inner spindle space was compromised and often appeared deformed with offshoots of the ER invading this space (Fig. 1G,H). Finally, unlike in control embryos, the ER did not efficiently elongate and separate into two separate populations during anaphase and telophase stages, suggesting the available space for the division was severely limited (Fig. 1D, arrowheads). As another means of visualizing ER morphologies, we used 2D embryo preparations (preps) in which embryos are punctured and isolated ER-nuclear figures can be examined under live-imaging conditions. These preps permit the enhanced resolution of ER behaviors, and we observed an increase in the density of ER tubules after *Rab1* disruption (Fig. S1E,F). Despite these changes in ER morphologies, *Rab1* disruption did not appear to activate stress indicators for the ER, such as XBP:GFP, a marker of the unfolded protein response (Fig. S1G). Further, despite ER morphology being linked to the cell cycle (Bergman et al., 2015), we did not find a difference in cell cycle timing in *Rab1-*disrupted embryos (Fig. S1H). These data demonstrate that the ER accumulates in close proximity to the incipient spindle and that a high level of ER accumulation after *Rab1* disruption is associated with a deformation of the inner mitotic space.

### *Rab1*-disrupted embryos display mitotic defects as a result of shortened ‘mini-spindles’

**Fig. 2.**
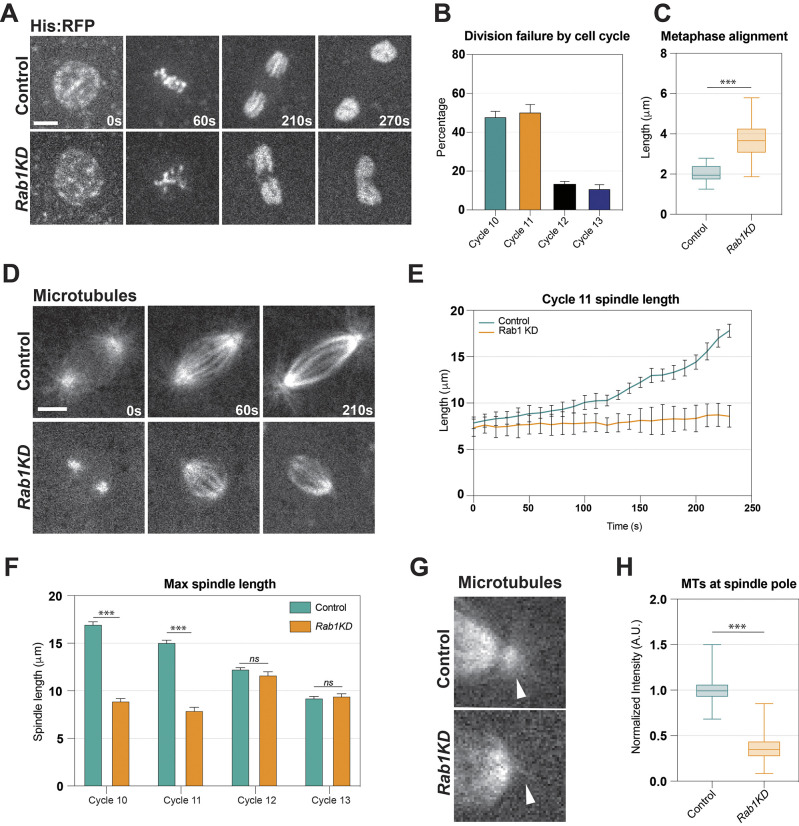
***Rab1* depletion causes mitotic division failures through the formation of a weakened ‘mini-spindle’.** (A) Still images from live imaging of His-2av:mRFP in prophase (0 s), metaphase (60 s), anaphase (210 s) and telophase (270 s) in control and *Rab1-*compromised (shRNA; *Rab1KD*) embryos. (B) Percentage of failed mitotic divisions in each cycle of the cortical syncytial divisions in *Rab1* embryos. k≥4 embryos; *n*=53 cycle 10, 145 cycle 11, 148 cycle 12, 110 cycle 13 measured mitotic figures. (C) Quantification of the alignment of chromosomes in metaphase based on the width of the apparent metaphase plate by histone fluorescence in control and *Rab1*-compromised embryos. k≥3, *n*=40. Measured at metaphase of cell cycle 11. (D) Still images from live imaging of spindle apparatus (Jupiter:GFP) in control and *Rab1*-disrupted embryos. (E) Spindle length over time in cell cycle 11 in control and *Rab1* embryos spanning from prophase to anaphase separation (measured every 10 s). k=3, *n*=10. (F) Maximum spindle length in control and *Rab1-*deficient embryos. k≥3, *n*=50. (G) Still images from live imaging of spindle pole microtubules (Jupiter:GFP) in control and *Rab1* embryos at metaphase in cell cycle 11. Arrowheads indicate location of centrosome. (H) Quantification of microtubules present at the spindle pole based on intensity normalized to control. k≥3, *n*=78. *Rab1* depletion through shRNA1 experiments; flies were maintained at 18°C prior to imaging at 25°C for stronger shRNA penetrance. ns, not significant; ***P<0.0005 (Mann–Whitney *U*-test). In box and whisker plots, box limits represent the inner quartile range, horizontal line represents the mean and whiskers the range. Scale bars: 5 µm. A.U., arbitrary units.

To examine further the effects of having an altered ER on mitoses in the early embryo, we imaged chromosomal segregation in control and *Rab1*-depleted embryos by expressing fluorescently marked histones (His:RFP). Consistent with the observed failures of ER to separate into two separate pools at anaphase (above), we found severe defects in mitotic function: 48% of mitoses failed in the first cortical division (cycle 10) and ∼50% experienced failure in the second cortical division (cycle 11) (Fig. 2A,B). Unlike other genetic interventions that disrupt divisions during the syncytial stages and are linked to the formation of the cytokinetic-like syncytial furrows, division failures were greatly reduced at cycle 12 (13.5%) and cycle 13 (11.1%) (Sullivan et al., 1993; Schejter and Wieschaus, 1993; Cao et al., 2010; Sherlekar and Rikhy, 2016; Xie and Blankenship, 2018). These defects in mitosis were consistent with two independent *Rab1* shRNA lines, indicating the *Rab1* specificity of the associated disruptions (Fig. S1I). Both shRNA lines were verified by qPCR, demonstrating *Rab1* depletions of 83-92% (Fig. S1J). We also wanted to examine more closely the nature of the mitotic failures – in previous work we observed that defects in mitoses could occur through either an inability to ‘corral’ or separate individual mitotic figures or through a ‘spindle collapse’ phenotype in which anaphase separation of chromosomes does not happen robustly (Holly et al., 2015). In *Rab1*-depleted embryos, chromosomes appeared to try to initially separate, but then collapsed back together and failed to divide into two individual populations, consistent with the spindle-collapse pathway (Fig. 2A, Movie 2). As defects in syncytial furrow formation can produce aberrant divisions by failing to separate or anchor mitotic spindles, we examined cortical F-actin intensities and furrow ingression behaviors in *Rab1-*depleted embryos (Sullivan et al., 1993; Schejter and Wieschaus, 1993; Riggs et al., 2003; Holly et al., 2015; Xie and Blankenship, 2018; Henry et al., 2022). Although *Rab1* furrows showed a mild decrease in furrow depths compared with control embryos, it appears unlikely that these small changes in furrow lengths explain the highly penetrant defects in mitoses as furrows still reached depths that can support successful mitoses and genetic stability (Xie and Blankenship, 2018; Fig. S2). We also found that actin intensities in both apical caps and furrows in cell cycle 10 and 11 were comparable to that of control embryos (Cao et al., 2010; Holly et al., 2015; Henry et al., 2022; Fig. S3). These combined data suggest that division failures in *Rab1* embryos are driven by an uncharacterized mechanism affecting chromosomal segregation.

In addition to the division failures, we noted a decreased ability of condensed chromosomes to align uniformly at the metaphase plate in *Rab1*-depleted embryos. Measurements of the width of aligned chromosomes nearly doubled in *Rab1* compromised nuclei that failed to divide (Fig. 2C). These above defects are potentially consistent with spindle function being compromised in *Rab1* embryos. We therefore wanted to examine spindle formation and structure directly in these embryos. To do so, we used a marker for microtubules (Jupiter:GFP, a microtubule-binding protein with GFP inserted at the endogenous locus) and imaged it in control and *Rab1*-compromised backgrounds. Strikingly, we found that during mitosis in *Rab1* embryos a ‘mini-spindle’ structure is formed in which the assembly and elongation of the spindle is deeply perturbed (Fig. 2D,E, Movie 3). Maximum spindle lengths are more than halved at cycle 10 mitoses (18 µm in control embryos and 7.5 µm in *Rab1* embryos). Interestingly, and similar to the observations of division failures, the maximum spindle length was significantly shortened in cell cycles 10 and 11, but cycles 12 and 13 were relatively unaffected after *Rab1* depletion (Fig. 2F). This concordance in the cycle-specific onset of these phenotypes is suggestive of a potential causal relationship between the formation of a weakened and shorter spindle and the segregation failures. Further, the spindles in *Rab1-*deficient embryos showed a markedly lower presence of microtubules at the centrosomes, with a greater than 50% reduction of Jupiter intensity at the spindle poles (Fig. 2G,H). Together, these data suggest that *Rab1* depletion causes early developmental defects that are highly penetrant in the division cycles at pseudo-cleavage stages. However, the division failures appear to be differentiated from the processes (such as F-actin and exocytic-driven furrow formation) implicated by previous works at the syncytial stages and therefore may be linked to the defective accumulation of ER in juxta-spindle regions.

### Mini-spindle phenotypes occur in conjunction with over-accumulated ER and are specific to ER disruption

**Fig. 3.**
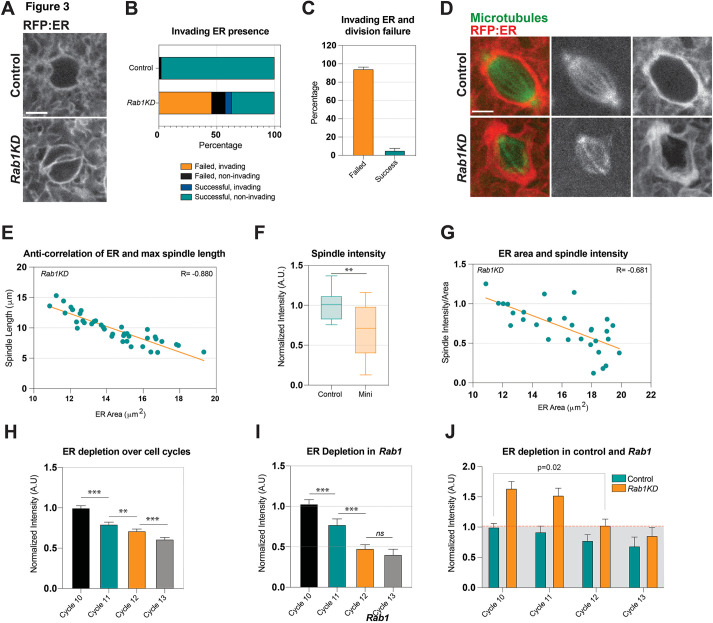
**ER accumulation correlates with a shortened spindle apparatus.** (A) Still images from live imaging of UAS-RFP:ER during prophase in control and *Rab1*-compromised (*Rab1KD*) embryos. Cell cycle 11 at z=2.5 µm from apical surface. (B) Incidence of ER intrusion on the inner-spindle space and whether mitoses fail or are successful in cell cycle 11. k≥7, *n*=138 mitotic events. (C) Division failures that have ER intrusion in cell cycle 11. k=8, *n*=92 mitotic events. (D) Still images from live imaging of the spindle (Jupiter:GFP) and ER (UAS-RFP:ER) at metaphase in cell cycle 11. (E) Anti-correlation of ER area and maximum spindle length measured from individual mitotic figures in cell cycle 11 of *Rab1-*disrupted embryos. Pearson R test shows negative (anti-) correlation. k≥3, *n*=40. (F) Spindle intensity plotted for control spindles and *Rab1* mini spindles at metaphase and normalized for spindle area. k=5, *n*=75. (G) Anti-correlation of ER area and spindle intensity (area-normalized) in *Rab1* embryos at cycle 11. Pearson R Test shows negative (anti-) correlation. k=3, *n*=32. (H) Mitotic ER intensities become depleted with each successive syncytial cycle in control embryos. Measured at metaphase and normalized to initial levels in cycle 10 embryos. k=7, *n*=51 cycle 10, 114 cycle 11, 132 cycle 12, 149 cycle 13. (I) Mitotic ER intensities also become depleted with each successive syncytial cycle in *Rab1* embryos. Measured at metaphase and normalized to initial levels in cycle 10 *Rab1* embryos. k=6, *n*=37 cycle 10, 88 cycle 11, 104 cycle 12, 133 cycle 13. (J) Comparison of ER depletion (intensity) in control and *Rab1-*depleted embryos normalized to cycle 10 of the control embryos. Dashed line indicates a potential ‘threshold’ above which ER accumulation inhibits spindle and mitotic function. ER channels in A and D were optimized as described in Fig. 1D. *Rab1* depletion was achieved through shRNA2 (Fig. S1I). ns, not significant; **P<0.005; ***P<0.0005 (Mann–Whitney *U*-test). In box and whisker plots, box limits represent the inner quartile range, horizontal line represents the mean and whiskers the range of the data. Scale bars: 5 µm. A.U., arbitrary units.

We next wanted to examine how directly related the defects in ER organization are with the ‘mini-spindles’ that form in *Rab1* embryos. We first measured the correlation between aberrant ER morphologies and division failures at the level of individual mitoses and found that in *Rab1*-disrupted embryos, the ER invades the mitotic space in ∼45% of division failures that occur in cell cycle 11 (Fig. 3A,B, Movie 4). Conversely, when divisions with ER invasion events were tracked, 94% resulted in a spindle collapse (Fig. 3C). Importantly, invading ER was not seen in control embryos in any instances, demonstrating the specificity of this phenotype to *Rab1* knockdown embryos. These data demonstrate a robust correlation in the incidences of these two phenotypes. We then used a two-color system to image the ER and spindle simultaneously under live-imaging conditions. These data revealed that the ER thickly packs around the spindle in *Rab1*-compromised embryos, with large aggregations of the ER surrounding the faintly present spindle poles (Fig. 3D, Movie 5). We also measured maximum spindle lengths and the amount of associated ER (µm^2^ area) and found a strong negative correlation in *Rab1* embryos (Pearson R=−0.880) (Fig. 3E). Microtubule spindle intensities (as judged by the Jupiter:GFP microtubule marker and normalized for spindle area) in these embryos were weaker, with mean intensities reduced by ∼30% (Fig. 3F). Similar to the measurements on spindle length, microtubule intensities in the spindle appeared to be negatively correlated with ER areas after *Rab1* disruption (Pearson R=−0.681) (Fig. 3G). We also examined potential explanations for why the later cortical cycles (cycles 12 and 13) do not possess the same degree of mitotic defects as in cycle 10 and 11 embryos by measuring mitotic ER accumulation during each syncytial cycle. These data showed that ER intensity decreases with each cycle of mitotic division in wild-type embryos (Fig. 3H), suggesting that the ER is depleted with each new round of mitotic divisions and furrow formation. A similar trend in ER intensities was observed in *Rab1* embryos (Fig. 3I). It is intriguing to note that the largest decrease in ER intensity in *Rab1* embryos was between cycles 11 and 12, which also correlates with when mitotic failures decrease (Fig. 3I). Additionally, although ER intensities were 64% greater in *Rab1* embryos at cycle 10 than in control embryos, owing to this cycle-dependent depletion they were only 8% greater during cycle 12 (compared with cycle 10 controls), potentially consistent with an ‘acceptable’ ER accumulation threshold for successful mitoses (Fig. 3J). These data demonstrate that the close association and accumulation of the ER after *Rab1* perturbation correlates with the failure to build a robust mitotic separation machinery.

**Fig. 4.**
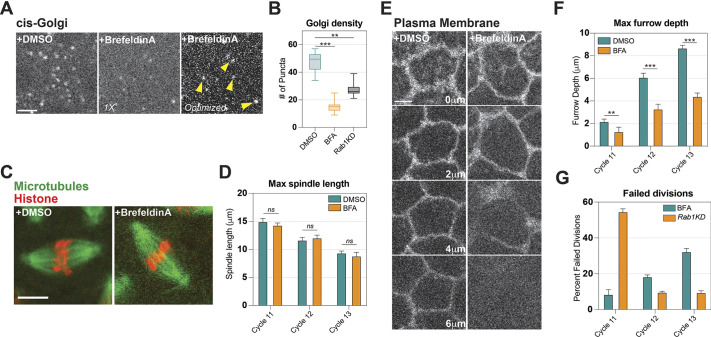
**Absence of early cycle mitotic or spindle lengthening defects after Golgi disruption.** (A) Still images from live imaging of cis-Golgi (Fws:GFP) after DMSO (control) or brefeldin A (BFA) injection. The right-hand panel shows a BFA-injected embryo with intensity levels optimized for visualization of Golgi puncta. (B) Density of Golgi compartments in control, BFA-injected, and *Rab1*-depleted embryos based on puncta number in a 25×25 µm region. k=6, *n*=24. (C) Still images from live imaging of miotic spindle (Jupiter:GFP) and chromosomes (Histone-2av:mRFP) injected with DMSO or BFA. (D) Quantification of maximum spindle lengths in syncytial cycles 11-13 of embryos injected with DMSO or BFA. k≥4, *n*≥40. (E) Still images from live imaging of Resille:GFP (plasma membrane) at different depths of syncytial furrow formation in DMSO- or BFA-injected embryos in cell cycle 12. (F) Maximum furrow depth at each cortical cycle in BFA- and DMSO-injected embryos. k≥4, *n*≥15 regions. (G) Percentage of failed divisions in BFA-injected embryos compared with *Rab1KD* (shRNA2) embryos. k≥5, *n*=104 cycle 11, 206 cycle 12, 319 cycle 13. ns, not significant; **P<0.005; ***P<0.0005 (Mann–Whitney *U*-test). In box and whisker plots, box limits represent the inner quartile range, horizontal line represents the mean and whiskers the range. Scale bars: 5 µm.

We also wanted to determine whether the mitotic defects and the failure to build a robust spindle after *Rab1* disruption were linked to decreased Golgi function. Indeed, as would be expected, *Rab1* depletion caused a reduction in Golgi size and number, although this reduction was somewhat modest in effect (Fig. S4A). As expected, both cis and trans Golgi compartments were similarly affected in *Rab1*-depleted embryos (Fig. S4B-D). To examine whether Golgi disruption could produce the ‘mini-spindles’ observed after *Rab1* disruption, we examined the effects of severely disrupting the Golgi by brefeldin A (BFA) treatment. BFA injection into embryos severely depleted Golgi densities, and to a stronger degree than that induced by *Rab1* depletion (Fig. 4A,B). Intriguingly, however, spindle behaviors appeared largely normal in the early cycles (Fig. 4C). Importantly, spindle elongation still occurred normally, and maximum spindle lengths were indistinguishable from those of control embryos at each measured cell cycle (Fig. 4C,D). Mitotic defects in the early cleavage divisions (cycle 11) also occurred at very low levels, unlike after *Rab1* disruption (8% after BFA injection in cycle 11 as opposed to 54% defective in *Rab1* embryos; Fig. 4G). In contrast to *Rab1* disruption, however, later cortical divisions in BFA-treated embryos began experiencing higher levels of failed divisions. This pattern of later cycle defects is the opposite of what occurs in *Rab1*-compromised embryos, but is similar to what is observed in embryos that have defects in building the deep syncytial furrows necessary for separating and anchoring mitotic figures (Cao et al., 2010; Mavor et al., 2016; Xie and Blankenship, 2018). Indeed, BFA embryos did not appear able to generate long furrows (Fig. 4E,F), consistent with a requirement for the rapid mobilization of an internal membrane pool in the construction of transient furrows at these stages (Sisson et al., 2000; Pelissier et al., 2003; Riggs et al., 2003; Fabrowski et al., 2013; Holly et al., 2015; Mavor et al., 2016; Miao et al., 2023). These data suggest that the mitotic defects and mini-spindles observed after *Rab1* disruption are due to the ER overaccumulation and not as a result of the relatively mild defects in Golgi size and number.

### Spindle shortening is correlated with a depletion of centrosome organizers and microtubule nucleators in *Rab1* embryos

**Fig. 5.**
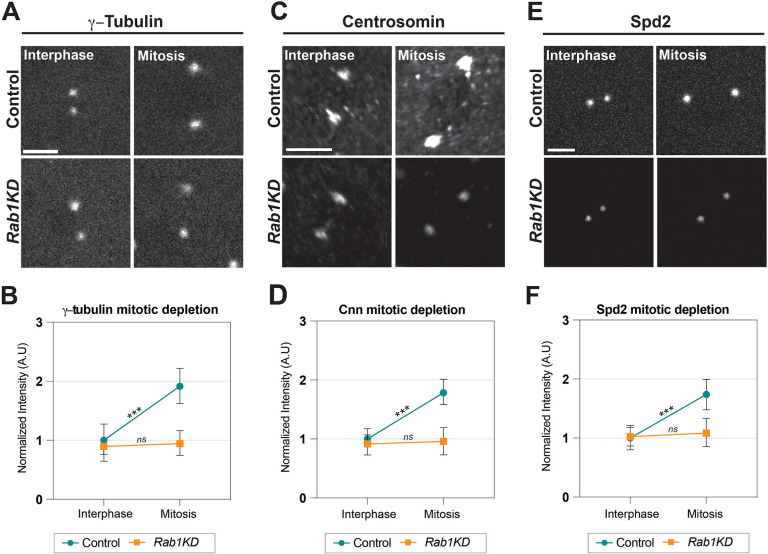
**Centrosomal maturation is inhibited in *Rab1*-disrupted embryos.** (A) Still images from live imaging of endogenously expressed γ-Tubulin:GFP in control or *Rab1* embryos at both interphase and mitotic time points. Mitotic measurements were taken at prophase (∼210 s in cycle 11). (B) Quantification of γ-Tubulin intensities at interphase and mitosis in control and *Rab1* embryos normalized to interphase of control. k≥4, *n*=44. (C) Images from fixed embryos stained with anti-Centrosomin in control or *Rab1* embryos at both interphase and mitotic timepoints. Mitosis was staged by DAPI stain. (D) Quantification of Cnn intensities at interphase and mitosis in control and *Rab1* embryos normalized to interphase of control. k≥4, *n*=45. (E) Still images from live imaging of endogenously expressed Spd-2:GFP in control or *Rab1* embryos at both interphase and mitotic timepoints. Mitosis measurements were taken at prophase (∼210 s in cycle 11). (F) Quantification of Spd-2 intensities at interphase and mitosis in control and *Rab1* embryos normalized to interphase of control. *Rab1* depletion was achieved through shRNA1 experiments; flies were maintained at 18°C prior to imaging at 25°C for stronger shRNA penetrance. k≥4, *n*=50. ns, not significant; ***P<0.0005 (Mann–Whitney *U*-test). Scale bars: 5 µm (A,E); 3 µm (C). A.U., arbitrary units.

To examine the mechanism responsible for weakened spindle function after ER disruption, we first determined whether *Rab1*-compromised embryos were able to recruit γ-Tubulin, a key protein in the spindle nucleating complex, γ-TuRC. Given the decreased spindle intensity observed in Fig. 3C,D, we hypothesized that microtubule nucleators may be depleted. Interestingly, we found that interphase pools of centrosomal γ-Tubulin in *Rab1* embryos were comparable to those of control embryos (Fig. 5A, left). However, centrosomes often undergo a maturation process at the onset of mitosis in which levels of microtubule nucleators are further enhanced (reviewed by Blanco-Ameijeiras et al., 2022). Consistent with centrosomal maturation operating in the early embryo, control centrosomes at cycle 11 almost doubled their γ-Tubulin levels by early in mitosis (Fig. 5A). Further, this process shows striking similarity to the timing of the ER concentration at the spindle poles (Fig. S5A). We therefore wondered whether this process of centrosomal maturation was defective in *Rab1*-compromised embryos. Indeed, although the interphase levels of γ-Tubulin were not significantly different, the further recruitment of γ-Tubulin during centrosomal maturation/mitosis was severely disrupted in *Rab1* embryos (control embryos increased by 91%, *Rab1* embryos by 4.9%; Fig. 5A,B), and mitotic figures that display division failure phenotypes also showed a depletion of γ-Tubulin (Fig. S5B). This was not due to a general decrease in γ-Tubulin protein levels in the embryos (Fig. S5C,D). The decreased recruitment of γ-Tubulin during mitotic spindle formation was intriguing. To study further the reasons behind this, we examined two upstream regulators of γ-Tubulin, Centrosomin (Cnn) and Spd-2. Previous work has documented Cnn as a key centrosomal protein, required for γ-Tubulin recruitment to the pericentriolar matrix (PCM) (Timothy et al., 1999; Zhang and Megraw, 2007). Similar to γ-Tubulin, Cnn intensities at the centrosome nearly doubled at the onset of mitosis (Fig. 5C,D), and this increase in maturation-associated intensities was also nearly absent in *Rab1* embryos (78% increase in control, 18% increase in *Rab1*). Although our data shows that Cnn recruitment follows similar trends as γ-Tubulin, some studies suggest that Cnn is not as essential in the formation of a functioning spindle (Megraw et al., 2001; Lucas and Raff, 2007). This prompted us to examine Spd-2, a centrosomal protein that aids Cnn in expanding the PCM (Conduit et al., 2014; Alvarez-Rodrigo et al., 2019). Spd-2 underwent a similar maturation in control embryos, and *Rab1-*compromised embryos also possessed a similar defect in Spd-2 recruitment at mitosis (∼63% decrease in maturation) (Fig. 5E,F), suggesting that PCM recruitment at mitotic stages is severely constrained in these embryos. Additionally, we found that spindle lengths in *Rab1-*depleted embryos correlated with Spd-2 intensity and that failed divisions overall showed weaker Spd-2 accumulation at the centrosome (Fig. S5E,F). Interestingly, Ana1, a protein associated with early centriolar to centrosome conversion, and the furthest upstream centrosomal regulator that we screened, showed similar levels between control and *Rab1* mitotic embryos, suggesting that ER overaccumulation does not affect centriole function (Fig. S5G,H). These results demonstrate that the timing of ER condensation/accumulation during mitosis in membrane-rich cleavage stages may pose challenges for the proper formation of the mitotic spindle and centrosomal maturation.

### Disrupting a Dynein-dependent mechanism of ER condensation during mitosis can rescue spindle function

**Fig. 6.**
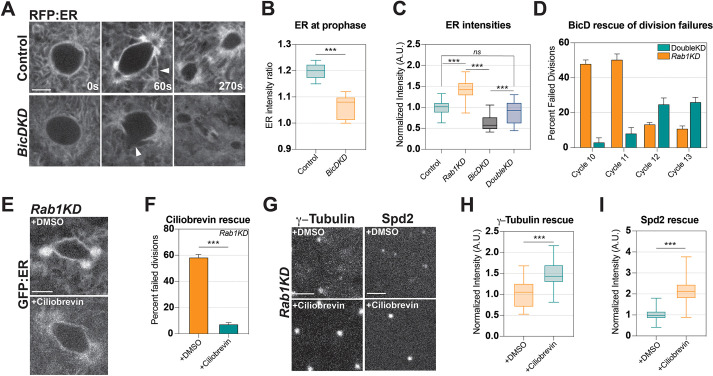
**Dynein disruption rescues *Rab1* mitotic failures and drives ER enrichment at the spindle poles.** (A) Still images from live imaging ER (UAS-RFP:ER) in control and *BicD*-compromised (shRNA; *BicDKD*) embryos marked at cycle 11. Arrowhead in control shows ER tubules and coat condensation. Arrowhead in *BicDKD* demonstrates local area of ER depletion adjacent to the inner spindle space. (B) ER condensation measured at mitotic entry in control and *BicD*-depleted embryos (ratio of perinuclear:cytoplasmic ER intensities). k≥4, *n*=70. (C) ER intensities at spindle poles in control, *Rab1* shRNA (*Rab1KD*), *BicD* shRNA and double *Rab1;BicD* knockdown (*DoubleKD*) embryos at metaphase*.* Normalized to control levels. k≥4, *n*≥55. (D) Division failure rates in *Rab1* embryos (orange) compared with double *Rab1;BicD* knockdown embryos (teal). (E) Injection of DMSO (control) and ciliobrevin D (80 µM) into *Rab1* embryos imaged with UAS-GFP:ER. (F) Division failure rates in cell cycle 11 when *Rab1* embryos were injected with either DMSO (control) or ciliobrevin D. k=6, *n*=168. (G) Still images from live imaging of *Rab1* embryos expressing γ-Tubulin:GFP or Spd-2:GFP injected with DMSO or ciliobrevin D at mitosis (∼210 s). (H) γ-Tubulin intensities in *Rab1* embryos during mitosis after control (DMSO) or ciliobrevin D injection (normalized to DMSO-injected control levels). k=4, *n*=80. (I) Spd-2:GFP intensities in *Rab1*-disrupted embryos during mitosis after control (DMSO) or ciliobrevin D injection (normalized to DMSO-injected control levels). *Rab1* depletion was achieved through shRNA2. k=4, *n*=60. ns, not significant; ***P<0.0005 (Mann–Whitney *U*-test). In box and whisker plots, box limits represent inner quartile range, horizontal line represents the mean and whiskers the range. Scale bars: 5 µm. A.U., arbitrary units.

The motor proteins that mediate the concentration of ER at the spindle poles and spindle coat in the early embryo have been unclear. Previous studies in spermatocytes suggested that Dynein regulates interphase ER localization to centrosomes, but largely focused on Dhc64c (Karabasheva and Smyth, 2019), which is also considered the major embryonic Dynein (Hays et al., 1994; Robinson et al., 1999). This cytoplasmic Dynein has been shown to localize to centrosomal and spindle areas during syncytial divisions as well as result in spindle defects when mutated, but its effect on ER localization remains unclear (Hays et al., 1994; Robinson et al., 1999). Additionally, there are eight Dynein heavy chains in the *Drosophila* genome, as well as a variety of intermediate and light family members, although many are implicated in spermatogenic function. Very few studies have focused on whether any of these Dynein family members effect ER organization, although they remain potential candidates for mediating ER-centrosomal localization. We were therefore interested in identifying the processes that localize ER near the centrosomes at mitosis in embryos. This would also permit the examination of whether the disruption of these centrosomal-enriching processes can rescue the mini-spindle phenotype after *Rab1* knockdown. To address this, we used two approaches to affect Dynein function broadly and approach potential genetic redundancies: genetic knockdowns of Dynein adaptor proteins and, to achieve acute disruption, injection of the Dynein inhibitor ciliobrevin D. We first examined embryos in which Bicaudal D (BicD), a protein that links the Dynein complex to its cargo in the embryo (reviewed by Hoogenraad and Akhmanova, 2016), function was compromised. Intriguingly, *BicD* shRNA embryos appeared to have less-robust mitotic accumulation of the ER, exhibited lower levels of spindle-adjacent tubules, and the coated structure surrounding the spindles was often disrupted (Fig. 6A, middle, arrowheads). This defect in ER accumulation was apparent at early mitotic stages, as ER intensities at prophase only increased by approximately half as much as in control embryos (Fig. 6A,B). Accumulation of the ER was inhibited throughout metaphase, when spindle pole intensities were depleted by ∼40% (Fig. 6C). shRNA-mediated BicD disruption was verified by qPCR (∼66% decrease; Fig. S6A). Thus, BicD disruption produces an opposite effect on ER morphologies during mitosis than that of *Rab1* disruption, in which a near equivalent increase in spindle pole ER intensities was observed (Fig. 6C). As an additional approach to disrupting Dynein function, we also compromised Dynactin activity, a major co-factor to Dynein, through expression of a Dynactin dominant-negative construct or by the overexpression of Dynamitin (also known as Dynactin 2, p50 subunit), an inhibitor of Dynein function (Burkhardt et al., 1997; Fig. S6B-E). In both cases, an ∼30% reduction of ER to the spindle poles could be seen at metaphase (Fig. S6C,E). These combined data suggest that Dynein, and Dynein-associated factors, mediate the condensation of the ER into tubular and near-centrosomal/spindle structures during the initiation of mitotic behaviors.

Based on these data, we hypothesized that whereas *Rab1* deficiency results in excess ER that inhibits spindle function and centrosomal maturation, disruption of Dynein in these embryos may block ER over-accumulation at the spindle poles and rescue division cycles. Indeed, in embryos that were deficient for both *Rab1* and *BicD* function*,* division failures in cell cycle 10 and 11 were reduced by ∼85-90% (Fig. 6D). To examine this rescue further, we used the Dynein inhibitor ciliobrevin D to disrupt Dynein function acutely at the syncytial stages. *Rab1* embryos expressing GFP-KDEL were injected with ciliobrevin D and scored for both ER morphologies and division failures (Fig. 6E,F, Movie 6). In these embryos, the aberrant accumulation of ER at the spindle poles and around the mitotic figures decreased, with the overall ER structure appearing more comparable to that in control (Fig. 6C,E). Additionally, ciliobrevin D-injected *Rab1* embryos displayed a rescue of apparent division failures, with the percentage of failed divisions in cell cycle 11 dropping from 58% in *Rab1* embryos to <10% after ciliobrevin D injection (Fig. 6F). We also investigated whether the mitotic rescue by Dynein inhibition was observable at the level of centrosomal function by examining γ-Tubulin and Spd-2 intensities (Fig. 6G). Here, we also observed that the efficiency of γ-Tubulin recruitment to the spindle pole increased by ∼40% after ciliobrevin D injection in *Rab1* embryos (Fig. 6H). Similarly, Spd-2:GFP exhibited an impressive doubling in intensity at the spindle poles compared with controls (Fig. 6I). Importantly, ciliobrevin D injection into control embryos expressing γ-Tubulin:GFP did not result in major changes to the recruitment of γ-Tubulin to the spindle poles, suggesting that the rescue is specific to *Rab1* disruption and not a non-specific additive effect (Fig. S6F). These combined results demonstrate that Dynein function plays an important role in directing ER morphologies during mitosis. Additionally, the ability of Dynein knockdown to partially rescue *Rab1* syncytial divisions suggests that centrosomal maturation defects are specifically linked to the altered ER localization and enrichment around the incipient spindles that occurs after *Rab1*-generated ER perturbation.

## DISCUSSION

**Fig. 7.**
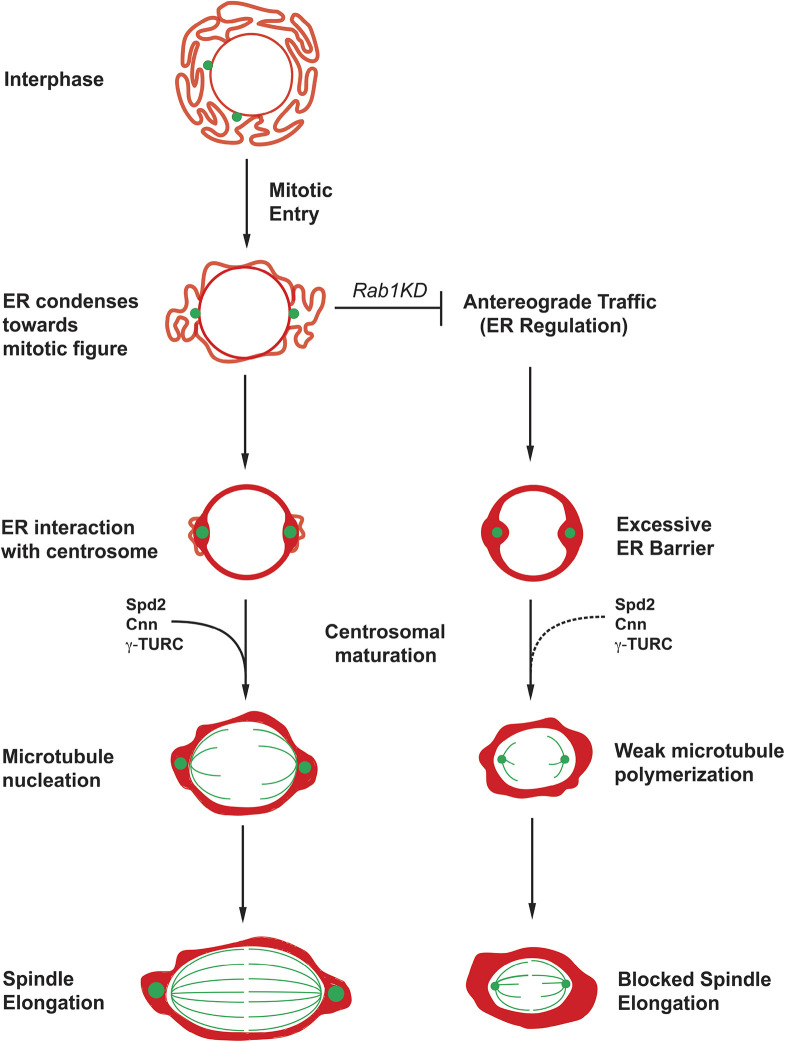
**ER dysregulation forms a barrier to PCM recruitment in *Rab1-*disrupted embryos.** In control embryos, ER morphological changes are coordinated with spindle behaviors. An initial interaction of the ER with centrosomes begins during prophase and is strengthened as spindle nucleation progresses. During this process, PCM recruitment is also occurring, allowing for the elongation of a robust spindle. However, disrupting ER morphologies through *Rab1* results in excess ER that inhibits centrosomal maturation, consistent with the ER acting as a barrier to PCM recruitment.

In early embryogenesis, multiple rounds of rapid cleavage divisions are necessary for the successful development of the organism across a variety of animal species. Although mitosis requires a myriad of dynamic structural changes to occur, the success of a division lies in the ability to elongate a robust spindle, capable of separating chromosomes into daughter nuclei. Here, we have shown that the careful tuning of ER morphologies contributes directly to proper spindle assembly and function in the *Drosophila* embryo. Disruption of the Rab GTPase *Rab1* generates an overaccumulation of the ER directly adjacent to the forming mitotic spindle. These ER defects inhibit the centrosomal machinery, which then nucleates a weaker ‘mini-spindle’ that is inefficient at the separation of duplicated chromosomes during anaphase. Decreased spindle size is highly correlated with increases in ER size or intensity. Interestingly, key microtubule nucleators and centrosome organizing proteins are depleted at the spindle pole in *Rab1-*compromised embryos, despite being present at similar cellular levels as controls, suggesting that accumulations of ER block the timely recruitment of these proteins (Fig. 7). Indeed, either genetic or small-molecule approaches to inhibiting Dynein function disrupted ER accumulation at spindle poles and subsequent mitotic failures were rescued. Combined, these results suggest an inherent conflict between the condensation of ER to the spindle poles, which has been implicated in the appropriate portioning of ER into resultant daughter cells, and the centrosomal maturation that strengthens centrosomes for spindle formation. This is a conflict that early cleavage-stage embryos may be particularly sensitive to owing to their highly enriched lipid environment.

Centrosomes are assembled around centrioles after their duplication, a process that does not seem to be defective in *Rab1* embryos, as the initial interphase appearance of the centrosomes appears to be unaffected. One of the earliest acting proteins in centriolar and PCM assembly that was examined in this study was Ana1; Ana1 intensities appear to be present at comparable levels in both control and *Rab1* knockdown embryos, suggesting that the defect is not associated with the early events in centriolar function. However, in many systems (Hamill et al., 2002; Kemp et al., 2004; Zimmerman et al., 2004; Kim and Rhee, 2014; Alvarez-Rodrigo et al., 2019; Blanco-Ameijeiras et al., 2022), centrosomes strengthen their microtubule-nucleating capabilities in preparation for mitosis and the formation of the microtubule spindle. This process of centrosomal maturation occurs in a similar time period as the inward ER condensation around the region where the spindle will form. Two major mediators of PCM function and the maturation of the centrosome are Spd-2 and Cnn (Timothy et al., 1999; Vaizel-Ohayon and Schejter, 1999; Zhang and Megraw, 2007; Lucas and Raff, 2007; Conduit et al., 2014; Alvarez-Rodrigo et al., 2019). Although the interphase recruitment of both proteins appears normal, their enhanced recruitment to the mitotic centrosomes during maturation is severely depleted in *Rab1* embryos. In turn, the decreased levels of spindle pole-associated γ-Tubulin and microtubules after *Rab1* disruption is an expected outcome, as Centrosomin and Spd-2 are required for the recruitment of γ-Tubulin and other microtubule-nucleating proteins that are key to spindle elongation (Timothy et al., 1999; Hamill et al., 2002; Kemp et al., 2004; Kim and Rhee, 2014; Zhang and Megraw, 2007). Thus, the depletion of these key centrosome proteins appears to be the causative agent in the generation of the short, weakened spindles found in *Rab1* embryos. It is also interesting to note that a recent study that analyzed dominant-negative expression of Atlastin, a protein implicated in the regulation of ER morphologies through homotypic fusion, also observed changes in ER structures that correlated with changes in spindle function in the fly syncytium (Araújo et al., 2022). Importantly, the authors observed a decrease in chromosomal separation velocities consistent with a weakened mitotic machinery after injection of dominant-negative Atlastin constructs. Thus, it appears that the ER in the early fly embryo plays an essential role in permitting the formation of a robust mitotic separation machinery.

Given this disruption of centrosomal maturation in *Rab1* embryos, what is the nature of the ER-associated spindle defect? In wild-type embryos, the ER appears to cradle centrosomes, whereas centrosomes in *Rab1* embryos appear to be closely impinged on by the large accumulations of ER. These aggregations are correlated with a weakening of centrosomal intensities (observed at multiple levels of microtubule, γ-Tubulin, and PCM-recruiting protein intensities). It is not clear at what scale this inhibition operates – it may be that the accumulated ER forms a dense enough network to form a diffusion barrier. Recent studies in *Caenorhabditis elegans* have suggested that the ER forms cradles around the centrosome as a filter for polymerizing microtubules (Maheshwari et al., 2023). Although this study suggests the filtering of materials exiting the ER cradle, this mechanism may also inhibit the entry of centrosomal regulators, especially in circumstances in which the ER cradle is aberrant or overly dense. Along similar lines, it has been shown in mammalian cells that vesicular trafficking carries γ-TuRC components to the centrosome during mitosis through Rab11-based trafficking (Hehnly and Doxsey, 2014), and thus the dense ER structures formed in *Rab1*-depleted embryos may impede endosomal routing to the centrosome. Interestingly, this same study suggested that Dynein activity was responsible for Rab11 movements to the centrosome; however, we did not observe a depletion of centrosomal intensities in embryos with compromised Dynein function, and instead saw a rescue of centrosomal and mitotic function only after co-depletion of Rab1 and BicD (or other disruptions of Dynein function). Lastly, we would note that laser-ablation experiments to cut the adjacent ER did not reveal elastic recoil (data not shown), suggesting that the ER does not physically constrain the spindle.

ER morphologies are highly choreographed in mitosis and show a clear organization in their compartmentalized zones within the embryo (Frescas et al., 2006). Although many ER proteins are potentially involved in maintenance of the structural integrity of the ER, the dynamic nature of the ER in the early embryos suggests a mechanism that promotes its rapid movement. Here, our results indicate that Dynein function is essential for ER accumulation at spindle poles. This is consistent with prior results from early *Xenopus* embryos and egg extracts (Wang et al., 2013). Data from *Drosophila* spermatocytes similarly observed a Dynein-driven enrichment of the ER to centrosomes just prior to the meiotic cell divisions, but also posited a potential Dynein-independent mechanism that operated during the later stages of meiosis (Karabasheva and Smyth, 2019). Previous studies have found that depleting Dynein can lead to the detachment of centrosomes from their nuclear-adjacent localization (Robinson et al., 1999); however, free centrosomes were rarely observed in our analyses of Dynein-disrupted backgrounds (*BicD* knockdown or ciliobrevin injection). Further work will be necessary to elucidate how Dynein aides in localizing the ER to the spindle poles in these stages, but various potential mechanisms are possible. Although a direct link of ER membranes or tubules to a Dynein motor cannot be ruled out, other non-direct interactions may be a major cause of this. One potential is ER ‘hitchhiking’ on a motor-associated vesicle in similar ways to other known hitchhikers, such as lipid droplets (reviewed by Salogiannis and Reck-Peterson, 2017). It is interesting to note that we also often observed interpolar ER bridges and malformations of the inner mitotic space after *Rab1* disruption, phenotypes that are consistent with a strong ER-microtubule/centrosomal interaction. It may therefore be that Rab1 trafficking is required, not just for tuning the overall amounts of ER prior to division, but, on a microscale. Rab1 or one of its interactors may also be essential to moderate the nature of ER-microtubule contacts. However, further work identifying Rab1 effectors and cargo will be needed to explore this topic further. In total, our work establishes that the ER and the mitotic separation machinery have a complex relationship in which the mechanisms that ensure the appropriate partitioning of ER during mitosis can also inhibit the same centrosomes that function in the construction of the mitotic spindle.

## MATERIALS AND METHODS

### Fly stocks and genetics

Fly stocks used in this study were: endogenous YFP:Rab1 (Dunst et al., 2015), UAS-Rab1 Val20 (BL-34670), UAS RFP:KDEL II (BL-30909), UAS-RFP:KDEL III (BL-30910) UAS-GFP:ER II (BL-59042), His2Av:RFP III (BL-23650), His2Av:RFP II (BL-23651), Jupiter:GFP Protein Trap (BL-60156), ncd-γ tubulin GFP (BL-57328), UAS-BicD Val22 (BL-35405), UAS-Xbp1:GFP (BL-39719). BL stocks were all obtained from Bloomington *Drosophila* Stock Center. Additionally, Spd-2:GFP (318743) was obtained from the Vienna *Drosophila* Resource Center, Fws:GFP (Farkas et al., 2003), Resille:GFP (A. Spradling, Carnegie Institution, DC, USA) and UASp-MoeABD:GFP (T. Millard, University of Manchester, UK) were also used. Rab1 Walium22 primers (shRNA2) were generated using the *Drosophila* Research and Screening Center protocol (https://fgr.hms.harvard.edu/knockdown-vectors) to establish a second, independent target sequence for *Rab1* RNAi. Primers (CTAGCAGTGAGTCTTTCAACAATGTGATAGTTATATTCAAGCATATCACATTGTTGAAAGACTCGCG and AATTCGCGAGTCTTTCAACAATGTGATATGCTTGAATATAACTATCACATTGTTGAAAGACTCACTG) were annealed and cloned into pWalium22 vector (*Drosophila* Genomics Resource Center stock 1473; https://dgrc.bio.indiana.edu//stock/1473; RRID:DGRC_1473) to create Rab1 Walium22. The construct was then verified through DNA sequencing. All fly crosses were maintained at 25°C except crosses containing Rab1 Valium20 (and corresponding controls) for maximal shRNA depletion; however, all imaging was performed at 25°C. BL-34670 Rab1 Valium20 (shRNA1) flies express a hairpin targeting the sequence TAGTGTAATAATGACGACATA. Rab1 Walium22 (shRNA2) was produced in lab, and expresses a hairpin targeting the sequence GAGTCTTTCAACAATGTGATAGTTA. Phenotypes were confirmed in both Walium and Valium lines. UAS flies were crossed to matαTub-Gal4VP16 67C;15 (D. St. Johnson, Gurdon Institute, Cambridge, UK) females to drive transgene expression.

### Live imaging, injection and 2D preps

Live imaging of embryos was carried out on a CSU10b Yokogawa spinning-disk confocal microscope from Zeiss and Solamere Technologies. Embryos were collected on apple juice agar plates with yeast and dechorionated in 50% bleach solution, washed with deionized water, then transferred to a slide with a gas-permeable membrane in Halocarbon 27 oil (Sigma-Aldrich). A coverslip was placed over the embryos for imaging. All images were obtained using a 63×/1.4 NA objective except for images of microtubules obtained for measuring spindle lengths, which were obtained with a 100×/1.25 NA objective. Images for initial characterization of phenotypes were obtained in 25-layer *z*-stacks at a 30 s imaging interval. Images used for precise time-resolved measurements were obtained in two-to five- slice *z*-stacks at 1-5 s intervals. All *z*-stacks were taken at 0.5 µm *z*-intervals between slices. For drug injections, embryos were glued to a coverslip after being dechorionated. The embryos were dehydrated in Drierite (stock 23001) for 20 min. Halocarbon 700 oil (Sigma-Aldrich) was placed on embryos prior to injection. Embryos were injected with 80 µM ciliobrevin D (Millipore Sigma, 250401) or 5 µg/ml brefeldin A (Santa Cruz Biotechnology, 200861) and imaged immediately following injection. To perform imaging on 2D preps, the vitelline membrane of embryos was gently pierced with sharp forceps and cellular contents allowed to spill out. 2D prep embryos were immediately imaged using the spinning-disk confocal microscope. Nucleus divisions were tracked to ensure that cell contents were still alive.

### Embryo fixation and immunostaining

Embryos were dechorionated with 50% bleach then washed with deionized water and manually devitilinized. Collected embryos were fixed at the interface of n-heptane and 4% formaldehyde in 0.1 M sodium phosphate buffer (pH 7.4) for 1 h. Embryos were then manually devitellinized and incubated with rabbit anti-Centrosomin (1:1000; J. Raff, University of Oxford, UK), anti-Ana1 (1:100; T. Avidor-Reiss, University of Toledo, OH, USA), mouse anti-GFP (1:500; Molecular Probes, A11120) or rabbit anti-GFP (1:500; Molecular Probes, A11122), anti-Golgin245 1:300 (Developmental Studies Hybridoma Bank, AB 2569587), anti-Lava Lamp 1:300 (lab of John Sisson, University of Texas at Austin, TX, USA). Primary antibody incubations were carried out overnight at 4°C with gentle agitation. Secondary antibodies conjugated to Alexa 488 or Alexa 568 (Molecular Probes, A11034, A11031) were used at 1:400 for 45 min. Secondary incubations were carried out at room temperature. Embryos were mounted on slides with Prolong Gold Antifade reagent +DAPI (Invitrogen, P36935) before imaging. Images of immunostained embryos were captured on an Olympus Fluoview FV1000 confocal laser-scanning microscope with a 60×1.35 NA objective.

### Intensity measurements and normalization

Intensity measurements were obtained from live imaging with spinning-disk confocal microscopy or from fixed and stained embryos (laser-scanning confocal). Embryos for intensity comparisons were imaged under identical imaging conditions as their controls (laser power, gain settings, exposure, etc.). In each imaged cycle, comparisons were set to the same *z*-layer, developmental time point, and fluorescence leveling as its respective control before obtaining intensity measurements. For intensity measurements, a region of interest was traced with freehand line or freehand area tool in ImageJ. ImageJ measurements of area, mean intensity values, max intensity values and min intensity values were taken for each trace. Background fluorescence was analyzed by tracing and obtaining measurements for a similar cytoplasmic length/area within the same *z*-layer and at the same time point. Five background measurements were averaged together, and average background was subtracted from the measured intensity. Obtained intensity values were then divided by mean intensity of controls to obtain a normalized value of fluorescence. For ER, Golgi and centrosomes, the area of fluorescence was traced using the freehand area tool in ImageJ and for furrow intensity the freehand line tool was used.

### Area and length measurements

Images for obtaining area measurements (compartmental and ER size, for example) were measured with the freehand selection tool on ImageJ. The perimeter of structures was traced, and areas measured in pixels. Pixel areas were converted to micron areas based on the pixel resolution of the microscope and objective used. For spindle lengths, the line tool was used to draw a single line across the spindle from pole to pole based on apparent centrosome location. Furrow depths were analyzed following the protocol previously described by our lab (Xie and Blankenship, 2018).

### ER tubule density measurements

Images of 2D-prepped embryos containing an ER marker were used to obtain ER tubule density measurements for control and *Rab1-*disrupted embryos. Images were obtained with identical microscope settings and leveled identically after imaging. Images were cropped into 150×150 pixel (25×25 μm) boxes and a diagonal line was drawn across the box. The number of tubules intersecting each diagonal line was counted to obtain a measurement of density of the tubules.

### Protein extraction, gels and western blotting

Proteins were extracted from embryos by collecting embryos for 2 h on apple juice agar plates. Embryos were dechorionated with 50% bleach solution, then washed and collected in a mesh net for control and *Rab1* embryos. An equal amount of embryos was transferred from the mesh net to Eppendorf tubes for each condition and manually ruptured in 50 μl of RIPA buffer [made in lab; Pierce RIPA Buffer (Thermo Fisher Scientific, 89900), 0.1 mM phenylmethanesulfonyl fluoride (Sigma-Aldrich, P7626-25G), 50 mM TCEP (Thermo Fisher Scientific, 20490) and Pierce Protease Inhibitor Tablet, 1 tablet in 10 ml (Thermo Fisher Scientific, A32955)]. Protein extracts were stored at −80°C. Resolving gels were prepared at 10% and stacking gels were prepared at 4%. Protein gels were run at 120 V for 1 h then transferred to PVDF membrane for 1 h at 100 V. Membrane was blocked in 5% skim milk and primary antibody incubation carried out at 4°C overnight. Antibodies used were: mouse anti-γ-Tubulin (1:3000; Thermo Fisher Scientific, MA5-31482) and mouse anti-neurotactin (1:100; Developmental Studies Hybridoma Bank, AB 528404). Secondary antibody incubation occurred over 1 h at room temperature using anti-mouse horseradish peroxidase (HRP) (1:10,000; Abcam, ab97023) and anti-rabbit HRP (1:3000; Abcam, ab205781) conjugated antibodies. HRP was developed using ECL reagent from Thermo Fisher Scientific (SuperSignal West PICO Plus, 34580) following the protocol for reagent use. Chemiluminescence images of blots were taken with the FluorChem R FR1030 system by ProteinSimple.

### qPCR of RNAi fly stocks

Embryos from flies expressing shRNAs against Rab1 and Bicaudal D were collected for 2 h on yeasted apple juice agar plates along with control embryos. Embryos were dechorionated in 50% bleach solution and transferred to an Eppendorf tube with a paintbrush. RNA was extracted using Zymo Research Quick-RNA MicroPrep Kit (Genesee Scientific, 11-327M) and stored at −80°C. RNA extraction was performed in triplicate for each biological condition. Reverse transcription reactions were carried out using the QIAGEN QuantiTect Reverse Transcription Kit (QIAGEN, 205311). The generated cDNA from each sample was used as template DNA for qPCR using the QIAGEN QuantiTect SYBR Green PCR kit (QIAGEN, 204143), and QIAGEN PrimerAssays (QIAGEN, 249900) for *sqh* (positive control), *Rab1*, *BicD* and *Rh3* (negative control) were used for qPCR experiments of the corresponding RNAi lines. qPCR was performed on a Bio-Rad iQ5 Multicolor Real-Time PCR Detection System and analyzed using the ΔΔ method to determine fold expression.

### Statistics and repeatability

All measurements represented in figures were obtained from imaging data of at least three embryos of at least three individual trials. In figure captions, k represents the number of embryos and *n* represents the number of individual measurements. Statistical significance was determined using the Mann–Whitney test, as denoted in figure legends. *P<0.05; **P<0.005; ***P<0.0005. Error bars indicate s.e.m.

### Image editing and figure preparation

Images obtained through spinning disk and laser scanning microscopy were edited using Adobe Photoshop. All images were leveled uniformly with their controls unless noted as ‘Optimized’ in the panel or caption. Optimized images were leveled to allow better visualization of structures when intensities were differentially affected in two backgrounds. Graphs were generated with Prism and figures were created in Adobe Illustrator.

## Supplementary Material

10.1242/develop.201917_sup1 Supplementary information
