# Supplementary material for: Dysregulation of the endoplasmic reticulum blocks recruitment of centrosome-associated proteins resulting in mitotic failure
Source: Development. 2023 Nov 16;150(22):dev201917. doi: 10.1242/dev.201917 (PMC10690056; doi:10.1242/dev.201917)
Supplement: Supplementary information [file develop-150-201917-s1.pdf]

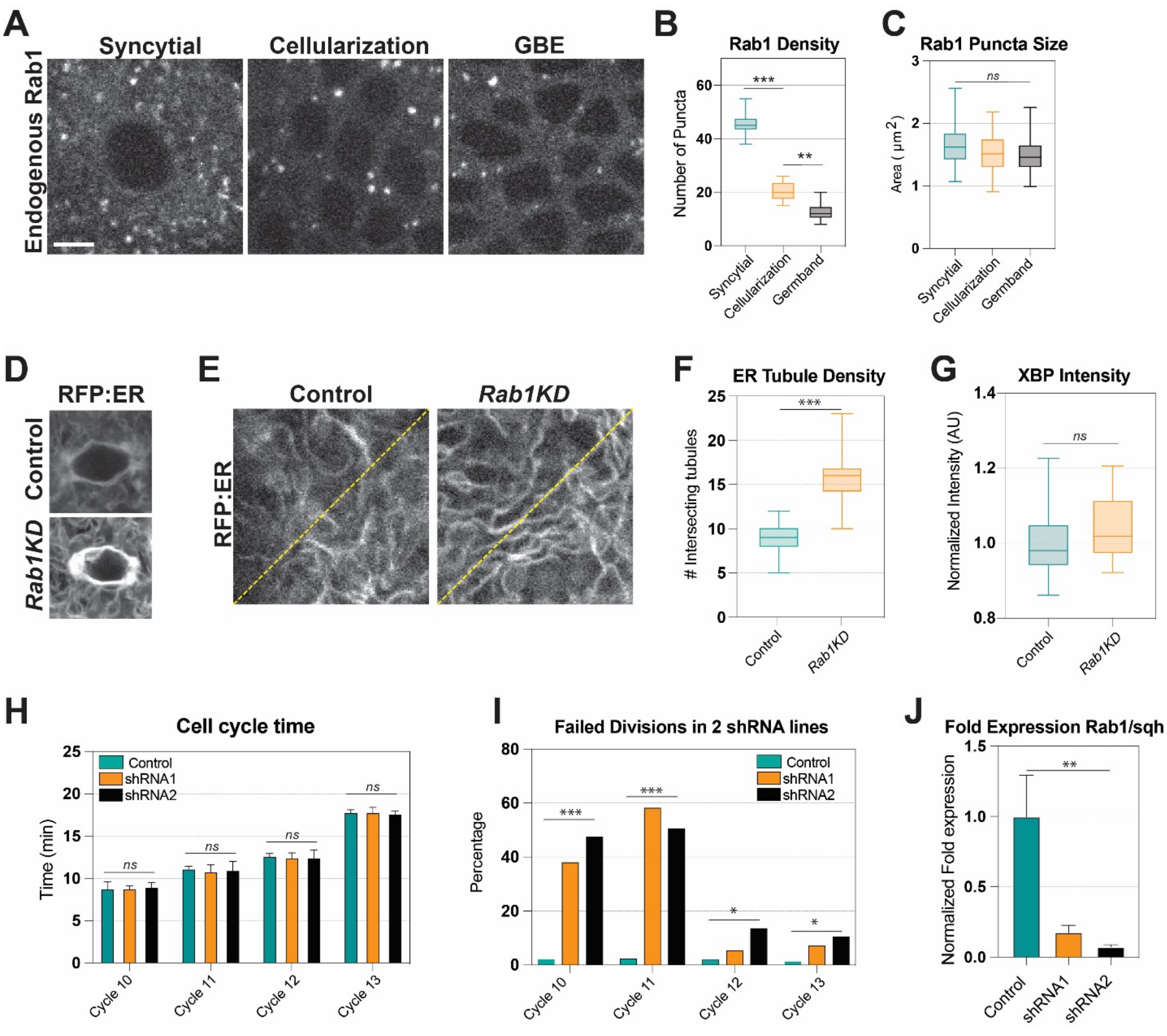

Fig. S1. Further characterization of Rab1 behaviors in embryos. (A) Still images from live imaging endogenously expressed YFP:Rab1 in early stages of embryogenesis. **(B)** Rab1 densities in each indicated stage of embryogenesis. Based on the number of puncta within a 25x25 µm box. k ≥3, n≥20. **(C)** Rab1 compartmental size (area) at each indicated stage of early embryogenesis. k≥3, n=100. **(D)** Representative images of ER in control and *Rab1* compromised embryos with identical intensity leveling. **(E)** Still images from live imaging of RFP:ER in 2D prepped (see Methods) control and *Rab1* disrupted embryos. Yellow line represents line drawn to count line-tubule intersections for tubule density measurement (F). **(F)** ER tubule density in control and *Rab1* embryos. k=6, n=32. **(G)** XBP:GFP (unfolded protein response marker) intensity levels in nuclei of control and *Rab1* deficient embryos. k=3, n=50. **(H)** Quantitation of cell cycle time in control embryos compared to cell cycle time in *Rab1* depleted embryos of 2

different shRNA lines k=3. **(I)** Percentage of division failures from two different *Rab1* shRNAs expressed in embryos. shRNA1= BL34670 Rab1 Val20 III, shRNA2= Rab1 Walium22 (created in lab, see Methods). k≥4. **(J)** Validation of Rab1 knockdown through qPCR in both shRNA lines used in the study. Rab1 expression was quantified against sqh as a housekeeping gene. *Rab1* depletion in A-G through shRNA2. ns= not significant; * = $p<0.05$; **= $p<0.005$; ***= $p<0.0005$. Statistics by Mann-Whitney U Test. Scale bar=5µm.

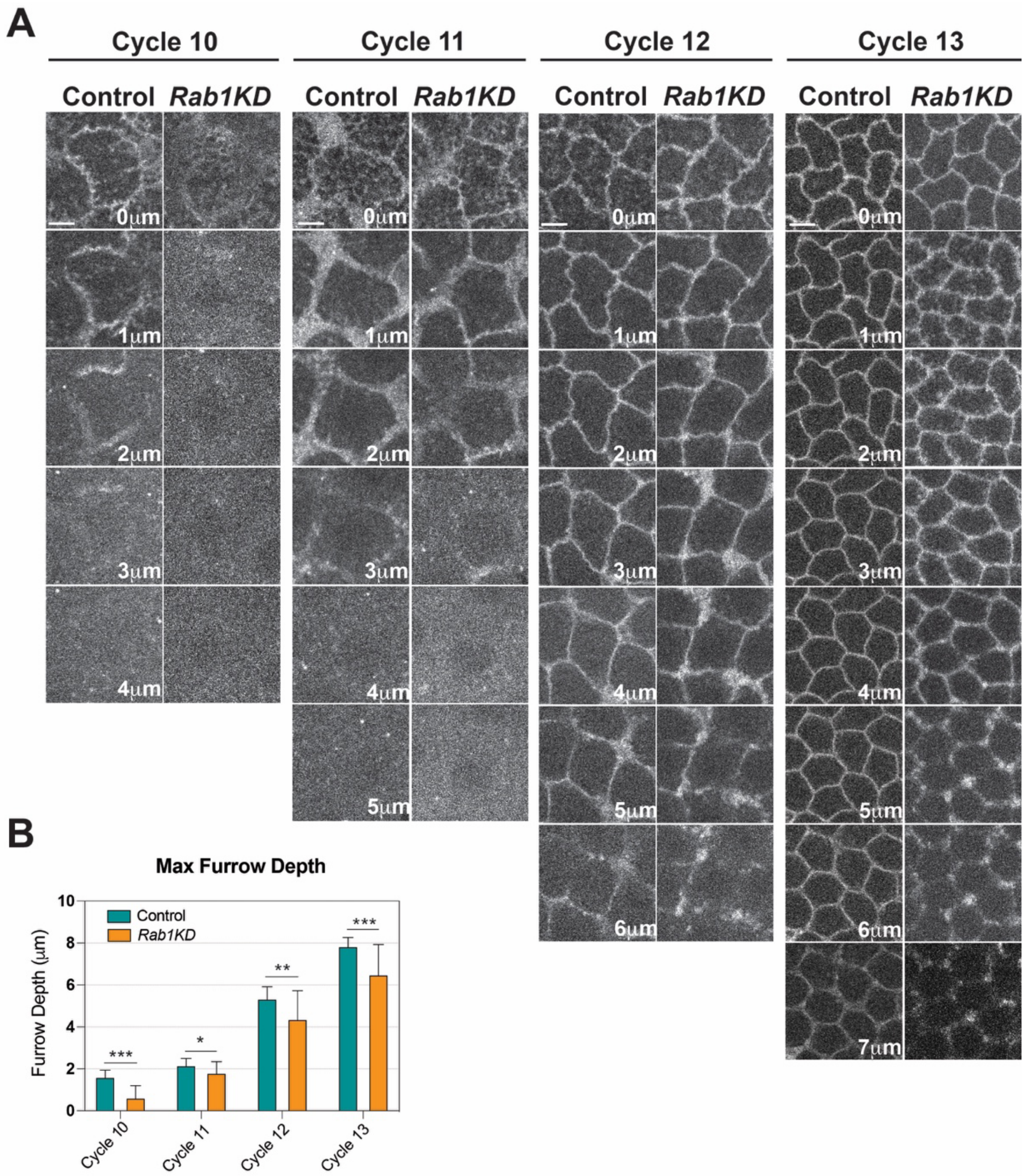

Fig. S2. Furrow formation in *Rab1* compromised embryos is mildly affected. (A) Still images from live imaging of membrane furrows (Resille:GFP) in control and *Rab1* embryos during each cortical division displayed at descending depths. **(B)** Quantitation of (A) showing average maximum furrow depth in each cell cycle. k≥3, n≥15. *Rab1* depletion through shRNA1. ns= not significant; * = p<0.05; **= p<0.005; ***= p<0.0005. Statistics by Mann Whitney U-Test. Scale bars=5µm.

Development • Supplementary information

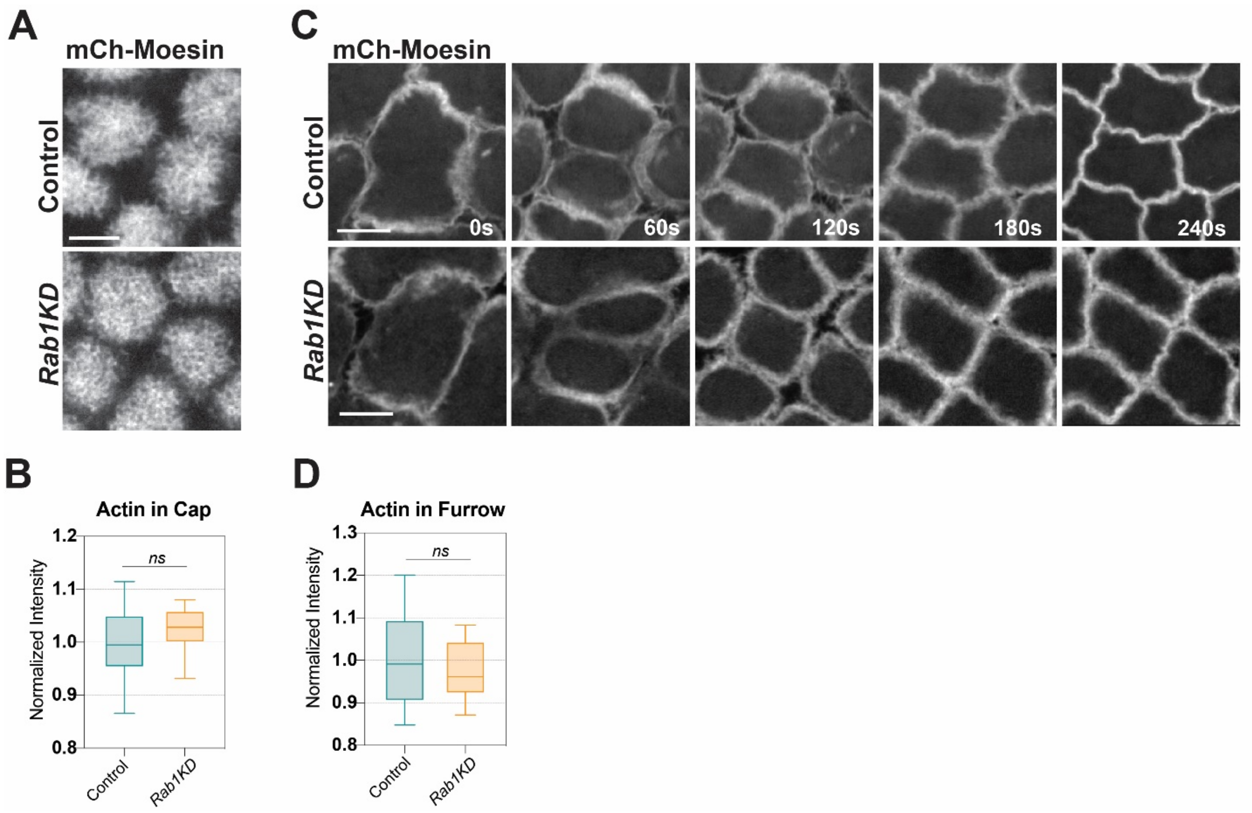

Fig. S3. Cortical F-actin levels are not disrupted after *Rab1* disruption. (A) Still images from live imaging of filamentous actin (marked by mCh:MoesinActinBindingDomain) in the apical actin cap in control and *Rab1* embryos at the same time point of cycle 11. **(B)** Quantitation of F-actin intensities in cap measured normalized to control. k≥3, n=55. **(C)** Still images from live imaging of mCh:MoesinABD in furrows at indicated time points during cycle 11.**(D)** Quantitation of F-actin intensities in the furrows measured normalized to the control intensities. k≥3, n=100. *Rab1* depletion through shRNA2. ns= not significant. Statistics by Mann Whitney U -Test. Scale bar=5µm.

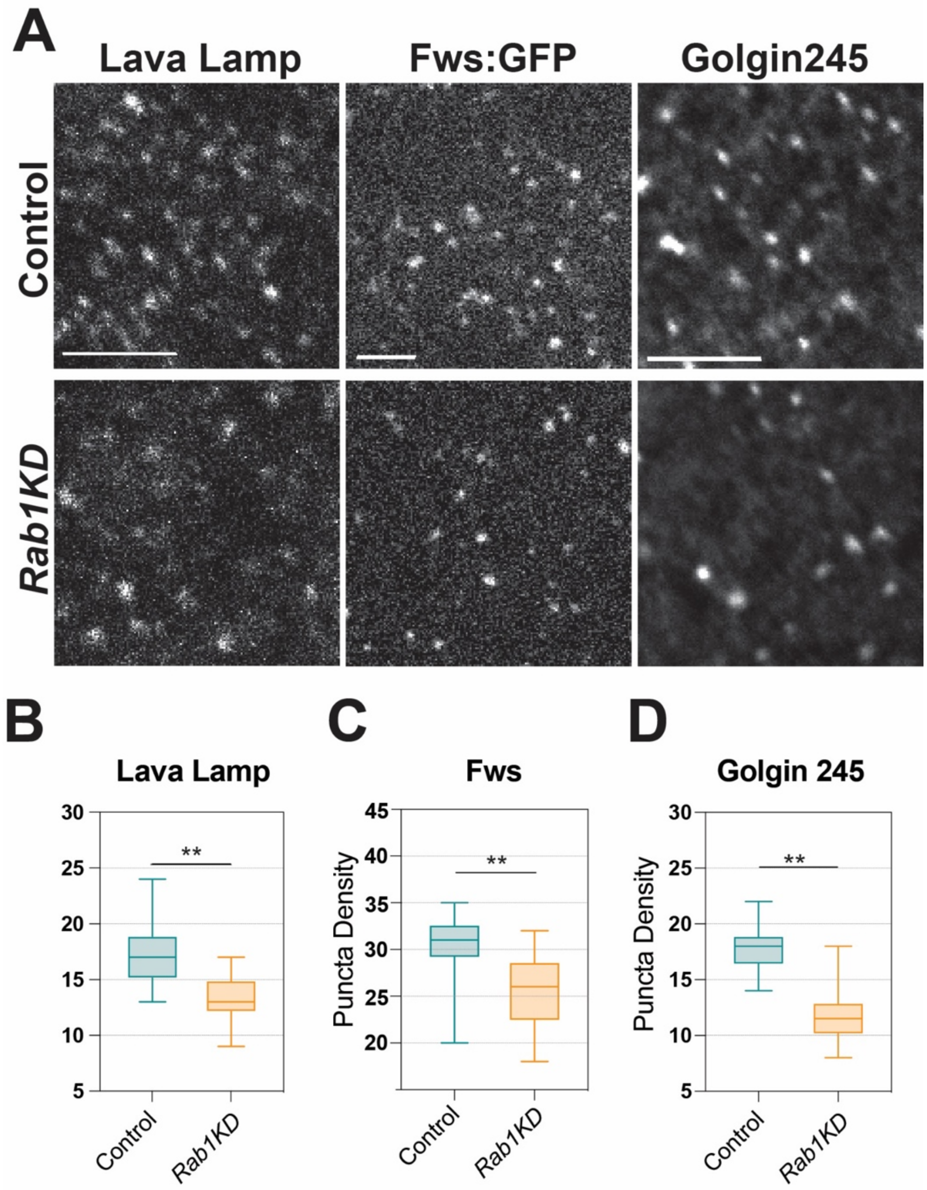

Fig. S4. Golgi characterization in control and *Rab1* depleted embryos. (A) Representative images from imaging of Golgi in live (Fws:GFP) or fixed (anti-Lava Lamp, anti-Golgin245) markers in control and *Rab1* shRNA embryos. **(B-D)** Quantitation of (B) anti-Lava Lamp (cis-Golgi), (C) Fws:GFP (cis-Golgi), and (D) anti-Golgin245 (trans-Golgi) number (densities) in control and *Rab1* disrupted embryos. Based on compartmental number in a 25x25µm box. k≥3, n=26 measured regions (Lava Lamp), n=41 measured regions (Fws), 22 measured regions (Golgin245). *Rab1* depletion through shRNA1. **= p<0.005. Statistics by Mann Whitney U Test. Scale bar=5µm.

Development • Supplementary information

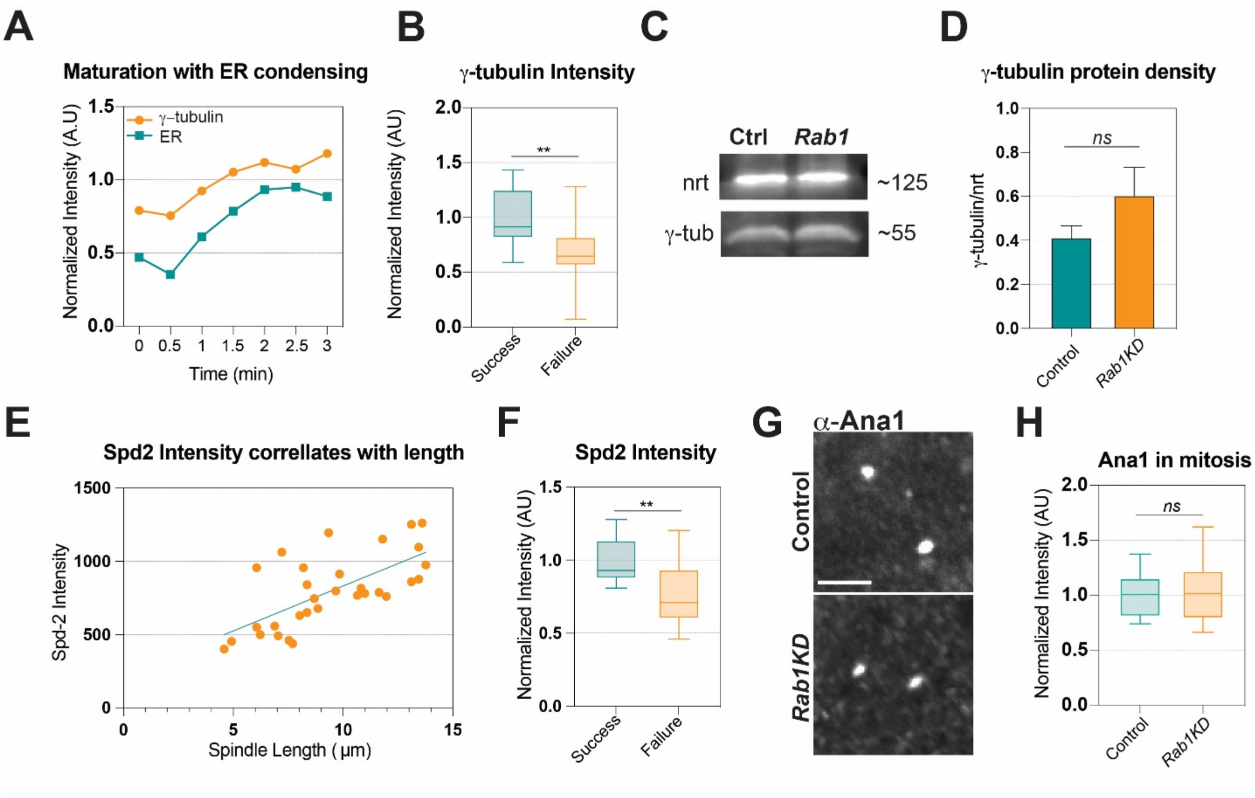

Fig. S5. Quantitation of various centrosomal behaviors. (A) γ-tubulin:GFP and ER (RFP:ER) intensities during the onset of mitosis (0=start of prophase) of syncytial cycle 11. k≥3, n=9 mitotic figures. **(B)** Quantitation of g-tubulin intensity in *Rab1* depleted embryos that successfully complete division compared to those that fail. k=3, n≥40. **(C)** Western blot of γ-tubulin and Neurotactin (loading control) levels in control and *Rab1* compromised embryos. **(D)** Quantitation of (B). n=3 western blots. **(E)** Quantitation of Spd-2GFP intensities in *Rab1* depleted embryos compared to spindle length shows correlation (Pearson R=0.6744). k≥4, n=35. **(F)** Quantitation of Spd2 intensity in *Rab1* depleted embryos that successfully complete division compared to that fail. k=3, n≥35. **(G)** Representative images from fixed embryos stained with anti-Ana1 in mitotic embryos in control and *Rab1* backgrounds. **(H)** Ana1 intensities in control and *Rab1* embryos. k=5, n=143. Analyses in cell cycle 11. ns= not significant; **= p<0.005. Statistics by Mann Whitney U-Test (except for western blot intensities (Student's T-test)). Scale bar=5μm.

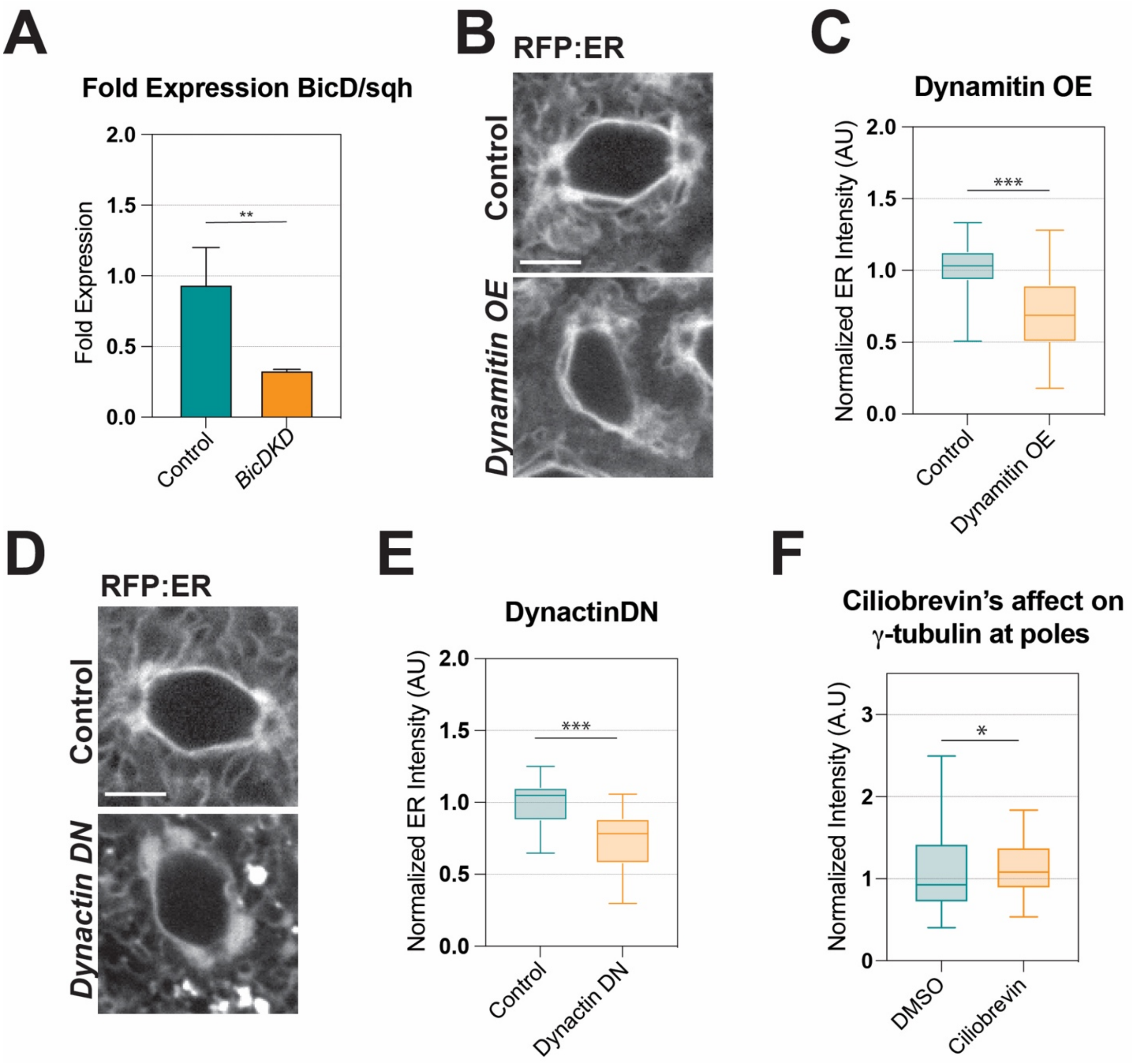

Fig. S6. Disrupting Dynein function shows depleted ER intensities at the spindle. (A) qPCR analysis of *BicD* depleted embryos shows depletion of BicaudalD when compared to sqh as a housekeeping gene. **(B)** Still images from live imaging of RFP:ER in control and Dynamitin overexpression embryos at metaphase. **(C)** Quantitation of ER intensity at the spindle pole in control embryos compared to dynamitin overexpression embryos during metaphase, normalized to control. k≥3, n≥45. **(D)** Still images from live imaging of embryos expressing RFP:ER in control and Dynactin dominant negative backgrounds at metaphase. **(E)** Quantitation of ER intensity at the spindle pole in control and Dynactin dominant negative embryos, normalized to control. k≥3, n≥40. **(F)** γ-tubulin intensities in control (DMSO) or Ciliobrevin D injected embryos. Rab1 is not disrupted. k=4, n=96. ns= not significant; * = $p<0.05$; **= $p<0.005$; ***= $p<0.0005$. Statistics by Mann-Whitney U-test. Scale bar=5µm.

Development • Supplementary information

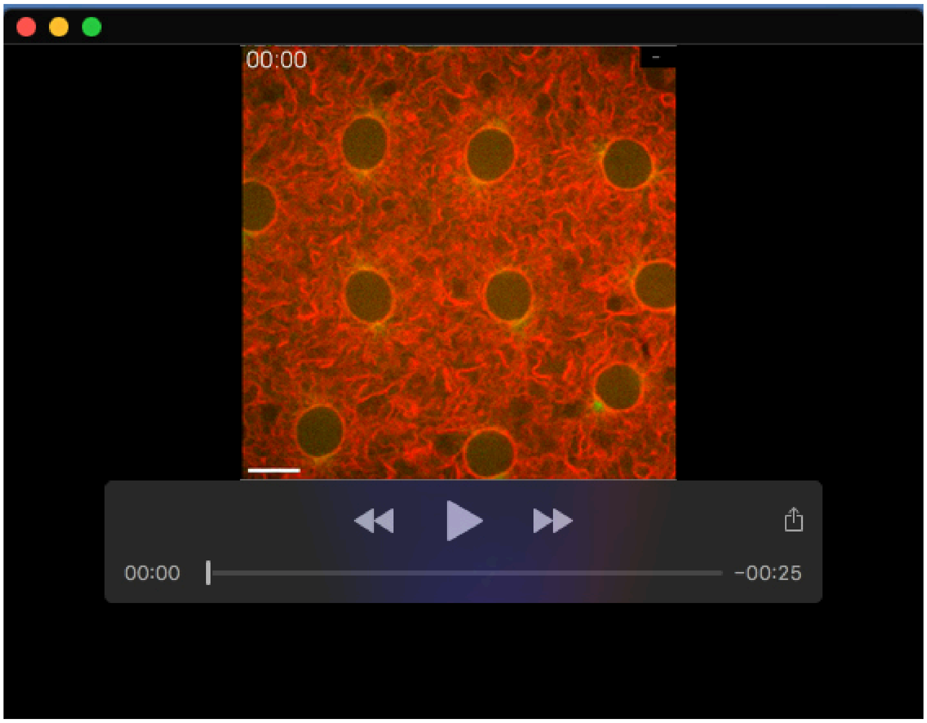

Movie 1. ER and Spindle display coordinated morphologies during mitosis. Time lapse imaging of the ER (UAS-RFP:ER) and spindle microtubules (Jupiter:GFP) during cortical divisions in the syncytial embryo. Images were collected at 30s time intervals and are displayed at 10 frames per second. Total developmental time= 47 min. Scale bar=10µm.

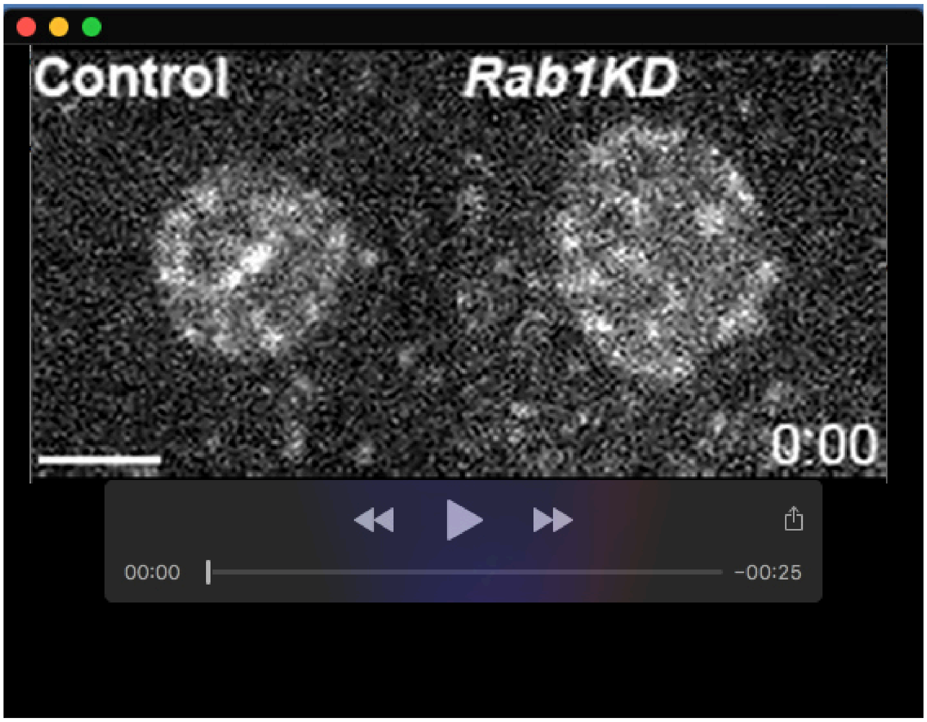

Movie 2. *Rab1* depletion results in spindle collapse. Time lapse imaging of representative nuclei (His-2av:mRFP) during cell cycle 11 in control and *Rab1* knockdown embryos. Images were collected at 30s time intervals and are displayed at 4 frames per second. Total developmental time= 6 min. Scale bar=5µm.

Development • Supplementary information

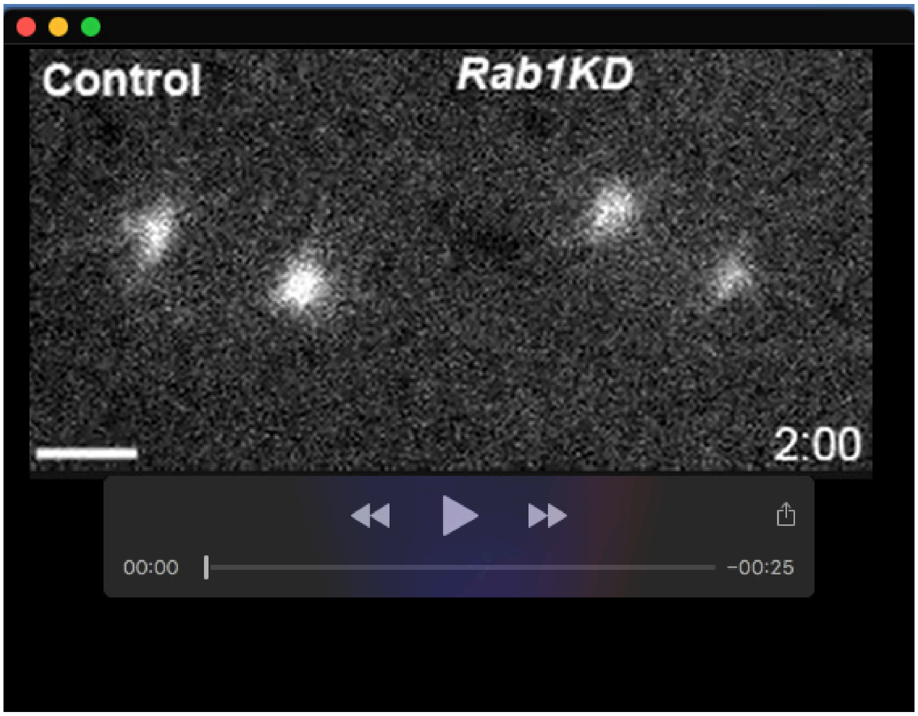

Movie 3. *Rab1* knockdown embryos display ‘mini-spindles’ that fail to elongate. Time lapse imaging of representative spindle (Jupiter:GFP) in control and *Rab1* depleted embryos. Images were collected at 30s time intervals and are displayed at 4 frames per second. Total developmental time= 5.5 min. Scale bar=5μm.

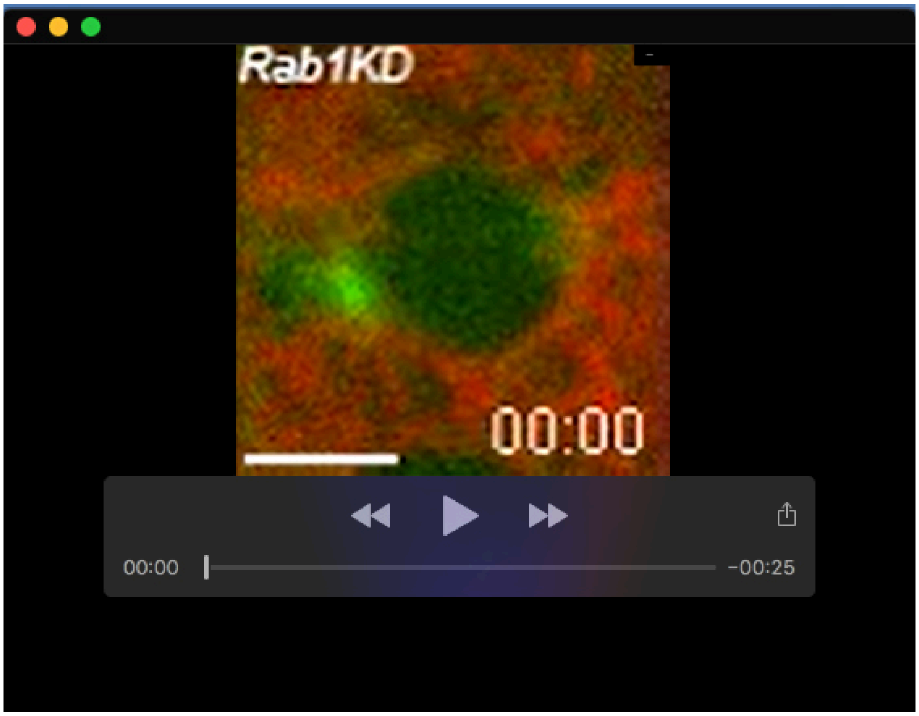

Movie 4. *Rab1* depleted inter spindle space displays invading ER in early mitosis. Time lapse imaging of ER (UAS-RFP:ER) and spindle microtubules (Jupiter:GFP) during cell cycle 11 in *Rab1* depleted embryo. Images were collected at 10s intervals and are displayed at 4 frames per second. Total developmental time= 2.5 min (150s). Scale bar=5μm.

Development • Supplementary information

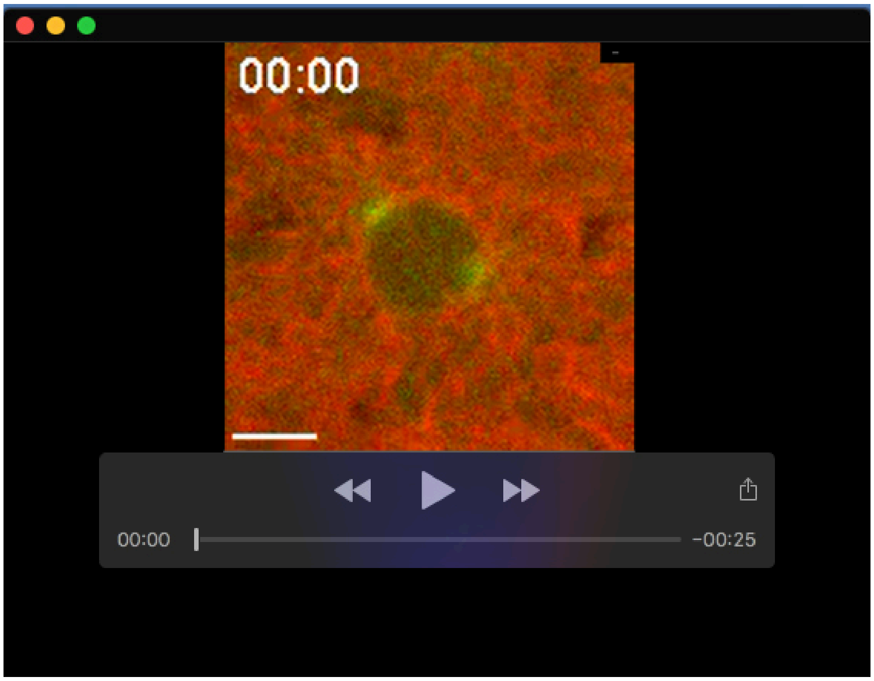

Movie 5. ER packing in *Rab1* depleted embryos results in shortened spindle. Time lapse imaging of the ER (UAS-RFP:ER) and spindle microtubules (Jupiter:GFP) in *Rab1* embryos during cell cycle 11 showing failed division and subsequent nuclear fallout. Images were collected at 5s intervals and are displayed at 10 frames per second. Total developmental time= 9.5 min. Scale bar=5µm.

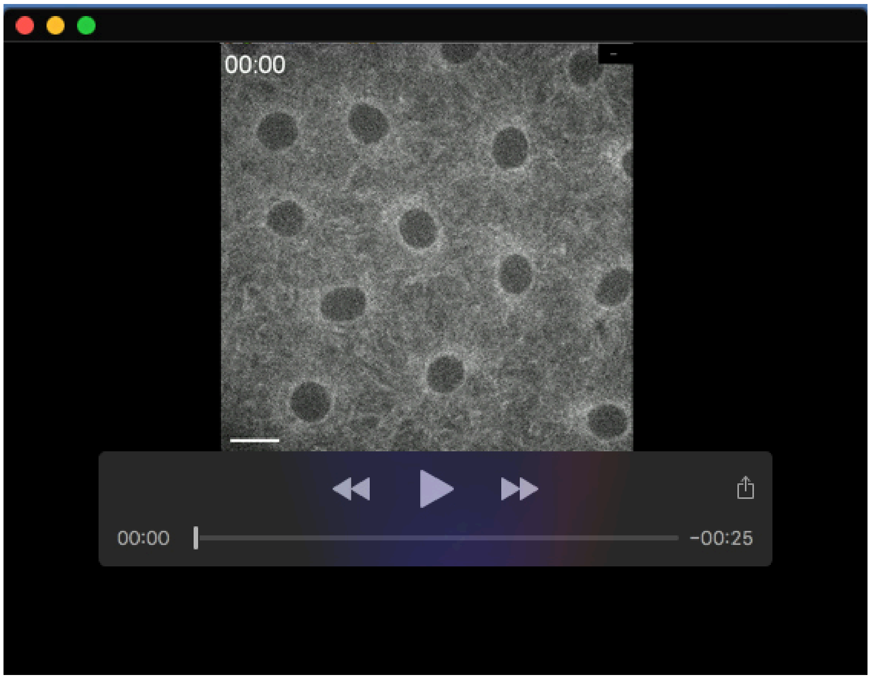

Movie 6. ER and mitotic phenotypes are rescued in Dynein inhibited *Rab1* embryos. Time lapse imaging of the ER (UAS-GFP:ER) in a *Rab1* depleted embryo injected with Dynein inhibitor, CiliobrevinD during cell cycle 11. Images were collected at 5s intervals and are displayed at 15 frames per second. Total developmental time= 9 min 10s. Scale bar=10µm.

Development • Supplementary information
